# Folates in Plants: Research Advances and Progress in Crop Biofortification

**DOI:** 10.3389/fchem.2017.00021

**Published:** 2017-03-29

**Authors:** Vera Gorelova, Lars Ambach, Fabrice Rébeillé, Christophe Stove, Dominique Van Der Straeten

**Affiliations:** ^1^Laboratory of Functional Plant Biology, Department of Biology, Ghent UniversityGhent, Belgium; ^2^Laboratory of Toxicology, Department of Bioanalysis, Ghent UniversityGhent, Belgium; ^3^Laboratoire de Physiologie Cellulaire Végétale, Bioscience and Biotechnologies Institute of Grenoble, CEA-GrenobleGrenoble, France

**Keywords:** folate, vitamin B9, biofortification, stress response, plant development, metabolism, methylation, neural tube defects

## Abstract

Folates, also known as B9 vitamins, serve as donors and acceptors in one-carbon (C1) transfer reactions. The latter are involved in synthesis of many important biomolecules, such as amino acids, nucleic acids and vitamin B5. Folates also play a central role in the methyl cycle that provides one-carbon groups for methylation reactions. The important functions fulfilled by folates make them essential in all living organisms. Plants, being able to synthesize folates *de novo*, serve as an excellent dietary source of folates for animals that lack the respective biosynthetic pathway. Unfortunately, the most important staple crops such as rice, potato and maize are rather poor sources of folates. Insufficient folate consumption is known to cause severe developmental disorders in humans. Two approaches are employed to fight folate deficiency: pharmacological supplementation in the form of folate pills and biofortification of staple crops. As the former approach is considered rather costly for the major part of the world population, biofortification of staple crops is viewed as a decent alternative in the struggle against folate deficiency. Therefore, strategies, challenges and recent progress of folate enhancement in plants will be addressed in this review. Apart from the ever-growing need for the enhancement of nutritional quality of crops, the world population faces climate change catastrophes or environmental stresses, such as elevated temperatures, drought, salinity that severely affect growth and productivity of crops. Due to immense diversity of their biochemical functions, folates take part in virtually every aspect of plant physiology. Any disturbance to the plant folate metabolism leads to severe growth inhibition and, as a consequence, to a lower productivity. Whereas today's knowledge of folate biochemistry can be considered very profound, evidence on the physiological roles of folates in plants only starts to emerge. In the current review we will discuss the implication of folates in various aspects of plant physiology and development.

## Introduction

Folates are indispensable components of metabolism in all living organisms (Bekaert et al., 2008). They play a role of donors and acceptors of one-carbon groups in one-carbon transfer reactions that take part in formation of numerous important biomolecules, such as nucleic acids, panthothenate (vitamin B5), amino acids. Supplying methyl groups for methyl cycle, folates are involved in methylation reactions that are not only of primary importance in regulation of gene expression, but are also necessary for the synthesis of lipids, proteins, chlorophyll and lignin.

Folates are synthesized *de novo* in bacteria, fungi and plants. Like other vertebrates, humans fully depend on their diet for folate supply. Being an important component of human diet, plants constitute the main source of folates for human population. Unfortunately, most staple crops such as potato, rice, cassava and corn are relatively poor in folates; hence, in regions where these staples are the main (or sole) energy source, folate deficiency is highly prevalent (Blancquaert et al., 2014). Insufficient consumption of folates was reported to be causally linked with various developmental defects and diseases, such as, neural tube defects and anemia. In addition, folate deficiency has been correlated with an increased risk for cardiovascular diseases, dementia and certain cancers (Blancquaert et al., 2010). In order to prevent the occurrence of such malignant disorders, enhancement of folate supplementation has been undertaken. Improvement of the nutritional value of staple crops is considered the most affordable and sustainable approach. Although significant success has already been achieved in the enhancement of folate content in a number of plant species, such as rice, tomato and lettuce (de La Garza et al., 2007; Storozhenko et al., 2007a; Nunes et al., 2009), some hindrances are to be tackled on the way to folate-rich crops. Indeed, biofortification strategies proved not to be equally efficient for different plant species, presumably due to differences in the regulation of the biosynthetic pathway. The inherent instability of the folate pool (Arcot and Shrestha, 2005; Quinlivan et al., 2006; De Brouwer et al., 2007) is another problem that has to be solved in order to prevent folate loss during post-harvest manipulations and storage. Moreover, in order to prevent possible interference with critical processes the effects of folate overproduction on overall plant metabolism have to be thoroughly studied.

Besides being essential for human health, folates are important for plant wellbeing as well. Thus, proper functioning of folate metabolism was demonstrated to be indispensable for plant development (Gambonnet et al., 2001; Ishikawa et al., 2003; Jabrin et al., 2003; Mehrshahi et al., 2010). Folates were reported to play important roles in signaling cascades (Stokes et al., 2013), as well as in nitrogen and carbon metabolism (Jiang et al., 2013; Meng et al., 2014). Folate supplementation was demonstrated to improve plant biotic stress resistance (Wittek et al., 2015). Moreover, folate metabolism was shown to be differentially regulated in response to various abiotic stress conditions (Baxter et al., 2007; Neilson et al., 2011), that pointed out its importance and possible specific adjustment in response to different stresses. Altogether these findings indicate that physiological roles and regulation of folate metabolism during development and stress response are important elements to be considered in the pursuit of crops with better productivity and improved stress tolerance.

In this review various aspects of folates in plants will be addressed, such as their chemical properties, biosynthesis, metabolic roles and turnover. Understanding of these aspects is a prerequisite to the development of a successful biofortification strategy. This paper also summarizes recent findings on the roles of folate metabolism in plant development and its link with other metabolic processes and signaling pathways, as well as points out the role of folates in plant stress response.

## Chemistry of folates

**Figure 1:**
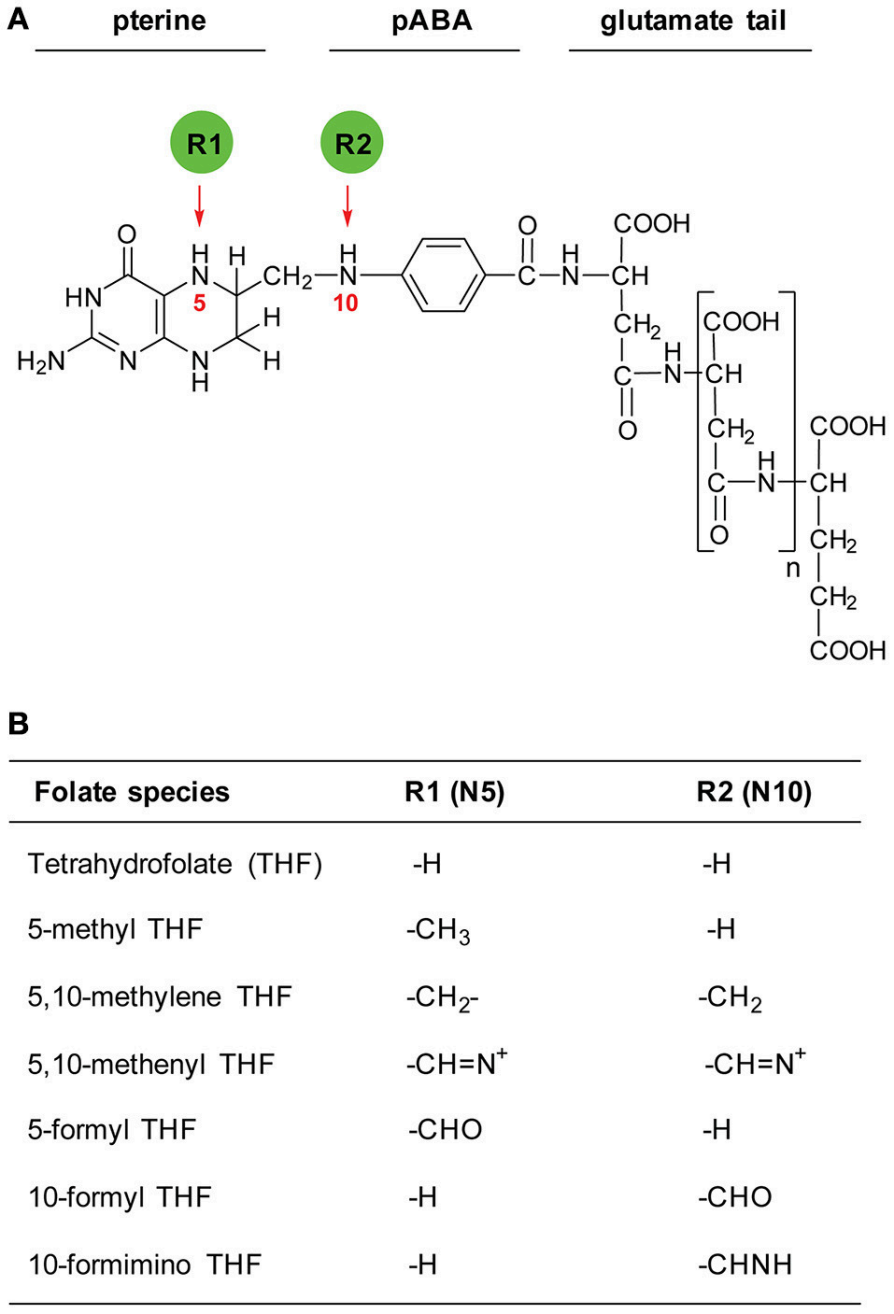
**Structure of THF and its derivatives. (A)** Structure of THF molecule. Red arrows indicate positions of one-carbon groups. R1 and R2 represent various one-carbon substituents. **(B)** Substituents carried by a THF molecule.

The term “folates” is generic for tetrahydrofolate (THF) and its derivatives. THF molecule is composed of three moieties: a pterin ring, a para-aminobenzoate (pABA) and a glutamate tail (Figure 1). Naturally occurring folates are dihydrofolate (DHF) and tetrahydrofolate (THF), which differ by the oxidation state of the pterin ring. While THF is a biologically active ready-to-use folate form, DHF requires reduction by dihydrofolate reductase (DHFR). A folate molecule with a fully oxidized pterin ring is called folic acid. Folic acid requires two rounds of reduction in order to become biologically active. THF molecules carry one-carbon units of various oxidation states attached to their N5 and/or N10 positions (Figure 1). The type of a one-carbon unit loaded onto a THF molecule determines its metabolic function. Folate molecules can also be distinguished by the number of glutamate residues that varies from 4 to 6 in plant naturally occurring folates.

## Folate biosynthesis in plants

**Figure 2:**
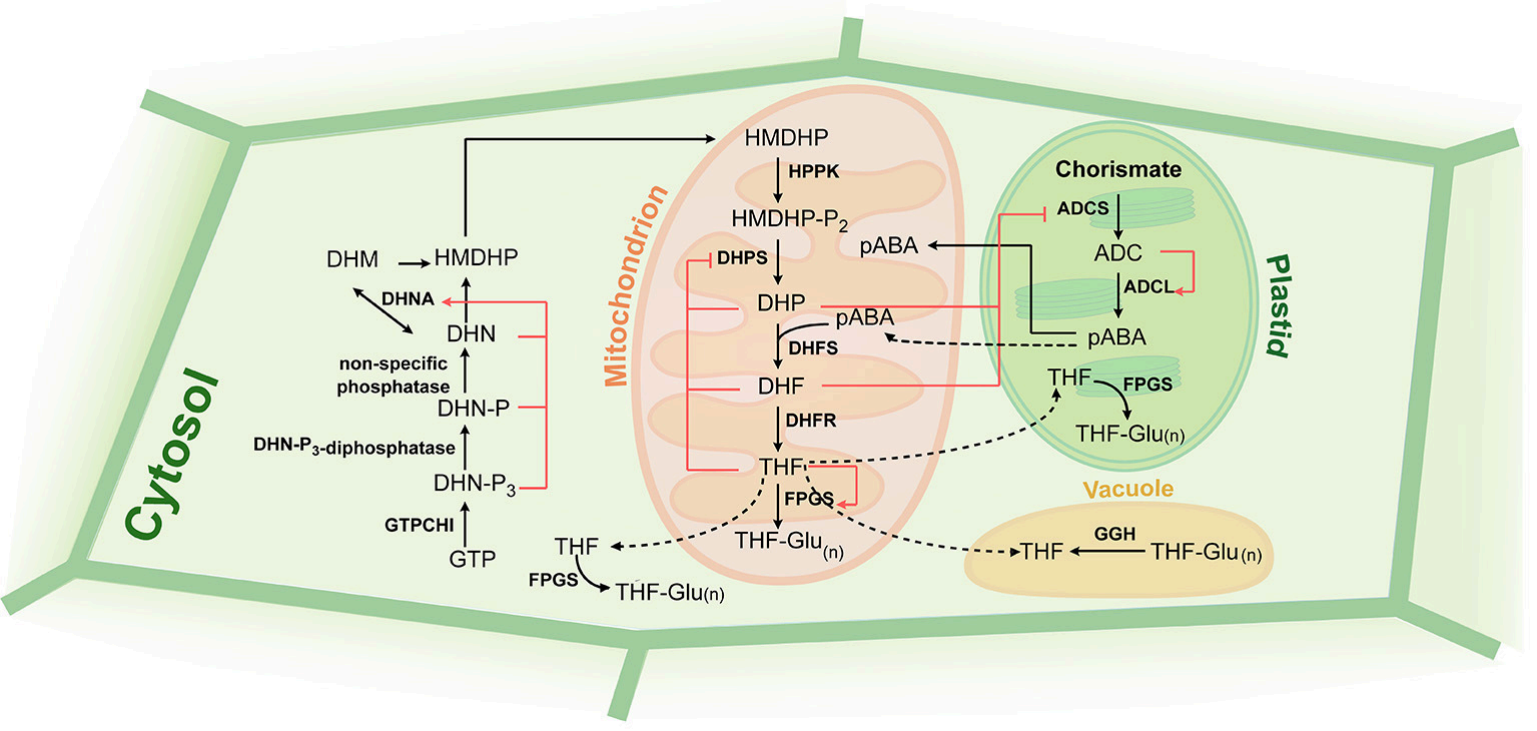
**Folate biosynthesis and regulation in plants**. Black arrows indicate biosynthetic steps, red arrows and blunt-end arrows show activation and inhibition, respectively, of enzymatic steps by folate precursors. Precursors: GTP, guanosine triphosphate; DHN-P_3_, dihydroneopterin triphosphate; DHN-P, dihydroneopterin monophosphate; DHN, dihydroneopterin; HMDHP, 6-hydroxymethyldihydropterin; HMDHP-P_2_, 6-hydroxymethyldihydropterin pyrophosphate; DHP, dihydropteroate; DHF, dihydrofolate; THF, tetrahydrofolate; THF-Glu_(n)_, tetrahydrofolate polyglutamate; ADC, aminodeoxychorismate; pABA, para-aminobenzoic acid. Enzymes: GTPCHI, GTP cyclohydrolase I; DHN-P_3_-diphosphatase, dihydroneopterin triphosphate pyrophosphatase; DHNA, dihydroneopterine aldolase; HPPK, HMDHP pyrophosphokinase; DHPS, dihydropteroate synthase; DHFR, dihydrofolate reductase; FPGS, folylpolyglutamate synthetase; ADCS, aminodeoxychorismate synthase; ADCL, aminodeoxychorismate lyase; GGH, gamma-glutamyl hydrolase.

While animals entirely depend on their dietary sources for the folate supply, plants, bacteria and fungi can synthesize folates *de novo*. In plants, THF biosynthesis is carried out in 11 steps and localizes to three subcellular compartments (Figure 2). Pterin and pABA moieties of THF molecule are synthesized in cytosol and plastids, respectively. Final five steps of the biosynthetic pathway are found to be mitochondrial and result in the production of polyglutamylated THF [THF-Glu_(n)_]. The biosynthetic steps are well-conserved among organisms-producers of folates. In the review we describe the thoroughly-characterized folate biosynthesis pathway in plants and draw a parallel with that in other organisms.

### Pterine synthesis in cytosol

#### GTPCHI

Synthesis of the pterine moiety starts with the conversion of GTP into dihydroneopterin triphosphate and formate, a reaction catalyzed by GTP cyclohydrolase I (GTPCHI). This conversion represents a rate-limiting step controlling metabolic flux into the folate pathway (Yoneyama and Hatakeyama, 1998; Hossain et al., 2004). GTPCHI from *E. coli*, yeast and mammals have been cloned and characterized (Nar et al., 1995; Nardese et al., 1996; Auerbach et al., 2000). Although mammals lack *de novo* folate biosynthetic pathway, GTPCHI is present in their genome and participates in tetrahydrobiopterin synthesis. *E. coli* and mammalian GTPCHI exist in a form of homodecamers comprising five tightly bound dimers. Studies on spinach, tomato, Arabidopsis and barrelclover (*Medicago truncatula*) demonstrate that plant GTPCHI has two tandem GTPCHI domains (Sohta et al., 1997). The tandem configuration of the proteins was shown to be an absolute requirement for their enzymatic activity, since the separate domains failed to exhibit any GTPCHI activity (Basset et al., 2002). The plant enzymes were shown to be cytosolic (Basset et al., 2002; McIntosh and Henry, 2008; McIntosh et al., 2008). The presence of GTPCHI transcript in developing seed tissues, leaves and roots in wheat suggested that *de novo* folate synthesis can occur throughout a plant body (McIntosh and Henry, 2008; McIntosh et al., 2008).

#### Dephosphorylation of dihydroneopterin triphosphate

Next, dihydroneopterine undergoes dephosphorylation that proceeds in two steps. The first step that is common in plants and bacteria is the removal of pyrophosphate by cytosolic Nudix hydrolase (Klaus et al., 2005b). The second dephosphorylation step is carried out by a non-specific phosphatase (Suzuki and Brown, 1974).

#### DHNA

The final step of the synthesis of the pterin moiety is catalyzed by dihydroneopterine aldolase (DHNA), which cuts the lateral side chain of dihydroneopterin releasing glycolaldehyde and 6-hydroxymethyldihydropterin (HMDHP) (Goyer et al., 2004). Plant DHNA activity is supported by three isoforms (Goyer et al., 2004) which, like their bacterial counterparts, are monofunctional DHNA enzymes (Güldener et al., 2004). In fungi and protozoa, DHNA activities were shown to be coupled with other enzymes of the folate synthesis pathway: with HPPK and DHPS in the former and with DHPS in the latter (Güldener et al., 2004). Due to the lack of obvious targeting signals, plant DHNA was defined as a cytosolic protein (Goyer et al., 2004).

### pABA synthesis in plastids

In plants, pABA is synthesized in pastids from chorismate in two steps (Nichols et al., 1989).

#### ADCS

First, chorismate and glutamine are converted to aminodeoxychorismate and glutamate in the reaction catalyzed by aminodeoxychorismate synthase (ADCS). Like its fungal (Edman et al., 1993; James et al., 2002) and protozoan (Triglia and Cowman, 1999) counterparts, plant ADCS exists as a bipartite protein with tandem domains homologous to PabA (the glutamine amidotransferase) and PabB subunits (the aminodeoxychorismate synthase) of *E. coli* ADCS (Basset et al., 2004a,b). In plants, it was shown that the NH3 released from the glutamine by the first domain is channeled toward the second domain to react with chorismate to form the aminodeoxychorismate (Camara et al., 2011).

#### ADCL

Second, aminodeoxychorismate is converted to pABA in the reaction mediated by aminodeoxychorismate lyase (ADCL) (Basset et al., 2004a,b). In *E. coli*, ADCL activity is supported by a monomeric PabC protein (Green et al., 1992), while in plants a homodimeric ADCL enzyme has been characterized (Basset et al., 2004a,b). Following its synthesis, pABA can be converted to its glucose ester by the activity of UDP-glucosyltransferase (Quinlivan et al., 2003; Eudes et al., 2008a). The pABA ester has no assigned function so far and is assumed to serve as a storage form of pABA. The esterification of pABA was demonstrated to occur at high rates in tomato fruits and leaves as well as in tissues of other plant species (Quinlivan et al., 2003). Assessment of the ADCL activity in various subcellular compartments revealed its predominantly cytosolic localization (Quinlivan et al., 2003). Owing to its amphiphilic nature, pABA is a membrane permeable compound and therefore can be freely distributed between subcellular compartments. Its esterification in cytosol restricts such passive transport into compartments and establishes a readily reclaimable storage form of pABA that can be shuttled to mitochondria by a dedicated transporter to enter folate biosynthesis (Quinlivan et al., 2003).

Both, ADCS and ADCL have been demonstrated to be feedback inhibited by high levels of pABA and its glucose ester, as well as by some folate species (THF, 5-methyl-THF and 5-formyl-THF) (Basset et al., 2004a,b).

### THF synthesis in mitochondria

#### HPPK/DHPS

The synthesis of THF in mitochondria starts with pyrophosphorylation of HMDHP and its subsequent coupling with pABA that results in the formation of dihydropteroate. These two reactions are catalyzed by HMDHP pyrophosphokinase (HPPK) and dihydropteroate synthase (DHPS) enzymatic activities. In *E. coli*, HPPK and DHPS are monofunctional enzymes (Dallas et al., 1992), while in plants (Rébeillé et al., 1997), protozoa (Triglia and Cowman, 1994), and fungi (Güldener et al., 2004) these two enzymatic activities are coupled on one protein. Fungal HPPK and DHPS are domains of a trifunctional protein possessing also DHNA activity (Güldener et al., 2004). In plants, DHPS is shown to be feedback inhibited by DHP, DHF, and THF (Mouillon et al., 2002).

#### DHFS

By the next step of THF synthesis a glutamate residue is attached to the carboxy part of the pABA moiety of dihydropteroate (DHP) to form dihydrofolate (DHF) in the reaction mediated by dihydrofolate synthetase (DHFS). Plant DHFS, like its fungal homolog, exists as a monofunctional enzyme. In bacteria, DHFS function is coupled with FPGS activity (Bognar et al., 1985).

#### DHFR

The penultimate step of the biosynthetic pathway is performed by dihydrofolate reductase (DHFR) and reduces DHF into THF. Like in protozoa, plant DHFR exists as a bifunctional enzyme coupled with thymidylate synthase (TS) (Luo et al., 1993; Neuburger et al., 1996; Cox et al., 1999), or as a monofunctional enzyme coupled with TS as a part of a multimeric complex (Toth et al., 1987). In bacteria, yeast and vertebrates, DHFR is a monofunctional enzyme. The enzyme was demonstrated to be localized in mitochondria and plastids in carrot and pea (Neuburger et al., 1996; Luo et al., 1997).

#### FPGS

The last step of the synthesis of folates is the attachment of a glutamate tail to THF molecule in the reaction catalyzed by folylpolyglutamate synthetase (FPGS). While in bacteria FPGS is coupled with DHFS enzyme, eukaryotes possess monofunctional FPGS. In Arabidopsis, three isoforms of FPGS exist and are targeted to three subcellular compartments: mitochondria, cytosol and plastids (Ravanel et al., 2001), which is in agreement with the presence of polyglutamylated folates in these compartments (Neuburger et al., 1996; Chen et al., 1997; Orsomando et al., 2005). A study of Arabidopsis FPGS double mutants, suggested that each individual isoform can localize to multiple compartments (Mehrshahi et al., 2010). Mammalian and fungal FPGS proteins are encoded by genes that produce both mitochondrial and cytosolic isoforms depending on the translation start site activated (Freemantle et al., 1995; DeSouza et al., 2000). Polyglutamylation affects compartmentalization of the folate pool by increasing anionic nature of folate molecules (Appling, 1991). Moreover, polyglutamylated folates are favored by folate-dependent enzymes over their monoglutamate forms (Shane, 1989). Hence, polyglutamylation can be regarded as an important regulatory point of the folate metabolism. FPGS activity is affected by the folate availability in the cell: high folate abundance inhibits FPGS activity in plants (de La Garza et al., 2007; Storozhenko et al., 2007a) and in mammals (Tomsho et al., 2008), while depletion of folate pool by the application of a folate synthesis inhibitor, methotrexate (MTX), results in an increase of FPGS activity (Loizeau et al., 2008). The regulation is assumed to occur on both transcript and protein levels (Loizeau et al., 2008).

## Folate salvage

**Figure 3:**
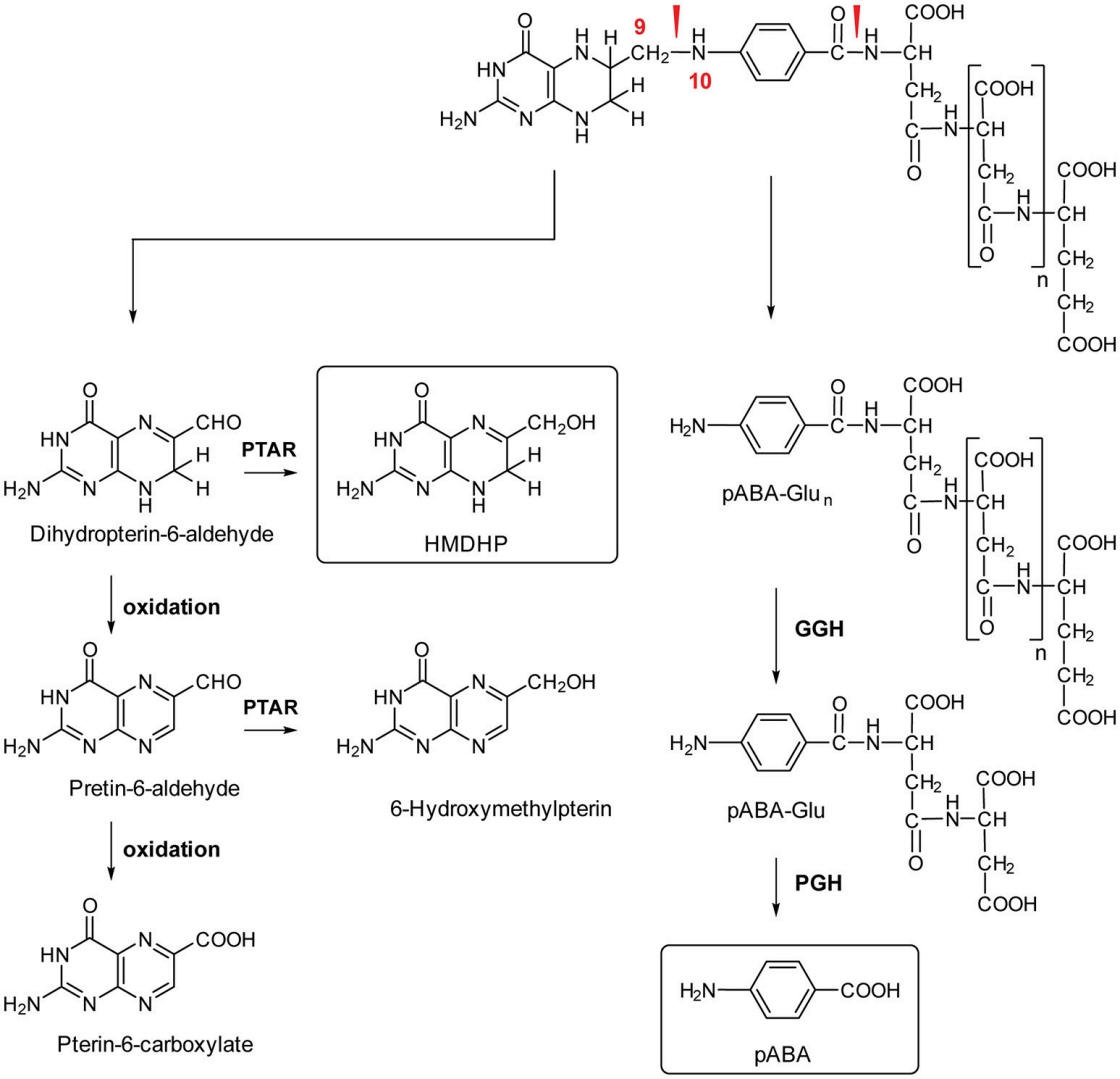
**Folate breakdown and salvage in plants**. Red arrowheads indicate bonds prone to oxidative cleavage. PTAR, pterin aldehyde reductase; GGH, gamma-glutamyl hydrolase; PGH, pABA-Glu hydrolase; HMDHP, 6-hydroxymethyldihydropterin; pABA, para-aminobenzoic acid.

Plants are continuously exposed to various stresses that result in elevation of reactive oxygen species (ROS) level—the cause of oxidative stress. Folates, being inherently unstable entities (Arcot and Shrestha, 2005; Quinlivan et al., 2006; De Brouwer et al., 2007), are extremely vulnerable to oxidative damage. Upon oxidation, DHF and THF undergo a non-enzymatic C9-N10 bond cleavage that yields a pterin tetrahydro- and dihydropterin-6-aldehyde, respectively, and para-aminobenzoylglutamate (pABAGlu) (Figure 3) (Gregory, 1989). Tetrahydro- and dihydropterin-6-aldehyde can be further converted to the fully oxidized aromatic form, pterin-6-aldehyde (Whiteley et al., 1968; Reed and Archer, 1980; Hanson and Roje, 2001). Non-enzymatic cleavage is assumed to be the main route of the folate break-down, although involvement of enzymes is not excluded (Scott, 1984; Suh et al., 2001).

Folates vary in their susceptibility to the cleavage. Thus, tetrahydrofolate and dihydrofolate are most vulnerable to degradation, while 5-formyl-THF and folic acid are most stable folate forms (Reed and Archer, 1980; Gregory, 1989).

Folate break-down rates are suggested to be high in plants. This notion is based on post-harvest studies of leaves and fruits that point to the rates of approximately 10% per day (Scott et al., 2000; Strålsjö et al., 2003). The same folate break-down rates of 10% were observed in the study on Arabidopsis plantlets supplied with an inhibitor of folate biosynthesis, sulfanilamide (Prabhu et al., 1998). Studies on mammals demonstrated that folate break-down products are excreted in urine (Scott, 1984), whereas fate of those in plants remains unclear. Some pieces of evidence suggest that the products can be re-used for folate synthesis. Thus, despite the high rate of folate break-down, pABAGlu and pterin moieties do not massively accumulate, as was demonstrated in the study on Arabidopsis and pea tissues (Orsomando et al., 2006). Moreover, *in vivo* and *in vitro* studies shown that folate break-down products are readily converted into folate precursors (Orsomando et al., 2006; Noiriel et al., 2007b).

Upon the breakage of the C9-N10 bond, pABAGlu/pABAGlu_n_ and pterine moieties are further recycled. The recycling of pABAGlu/pABAGlu_n_ starts with an enzymatic cleavage of the Glu tail, if any present, mediated by gamma-glutamyl hydrolase (GGH) (Figure 3) (Akhtar et al., 2008, 2010). The enzyme is located in vacuoles (Orsomando et al., 2005; Akhtar et al., 2008). Despite a very high GGH activity, polyglutamylated folates still exist in vacuoles. Two scenarios can be suggested to resolve this paradoxical co-existence. First, it is possible that folate polyglutamates are stabilized by folate binding proteins (FBP), as it was shown to occur in mammals (Hutchinson et al., 2000; Jones and Nixon, 2002). Plausibility of this hypothesis was demonstrated by a recent study showing an enhancement of folate pool stability in rice grains by expression of mammalian folate binding protein (FBP) (Blancquaert et al., 2015), although, the existence of plant FBP remains to be demonstrated. Second, polyglutamylated folates might be sequestered away from highly active GGH in vacuoles (Orsomando et al., 2005).

In the next step, pABA is released from the Glu by the activity of pABAGlu hydrolase (PGH). Although plant PGH genes have not been identified yet, PGH activities were found in Arabidopsis, pea and tomato (Orsomando et al., 2006). Studies on Arabidopsis and pea detected PGH activity in mitochondria, cytosol and vacuoles and suggested existence of at least two isoforms of the enzyme (Bozzo et al., 2008), which is in agreement with the finding of two isoforms of the enzyme in *E. coli* (Hussein et al., 1998).

The recycling of the pterin cleavage product, dihydropterin-6-aldehyde, is achieved through its reduction to HMDHP, the folate precursor (Orsomando et al., 2006). The reduction was shown to be performed by non-specific pterin aldehyde reductase (PTAR) (Noiriel et al., 2007a,b). In pea, PTAR activity was found to localize mainly in cytosol (at most 1% of the total activity is detected in mitochondria) and to be contributed by several isoforms (Noiriel et al., 2007a,b). One of the PTAR isoforms was found to be encoded by At1g10310 gene in Arabidopsis. The predominance of PTAR localization in cytosol suggests that the break-down of pterin products (pterin aldehydes) must be transported from plastids and mitochondria to cytosol, in order to be recycled. It is possible that a dedicated transporter conducting this shuttling exists. Dihydropterin-6-aldehyde that did not fall under recycling can be subjected to further oxidation to pterin-6-aldehyde, the fully oxidized form of the aldehyde. Plants lack the capacity to recycle the fully oxidized pterin aldehyde (Noiriel et al., 2007a,b). To date, only Leishmania is known to reduce the fully oxidized pterin aldehyde (Bello et al., 1994). The reduction is performed by PTR1 enzyme that converts pterin-6-aldehyde to tetrahydropterin-6-aldehyde through 7,8-dihydro- state in a two-stage NADPH-dependent reaction (Bello et al., 1994). The lack of PTR1 activity in plants implies that the fully oxidized pterin aldehyde cannot be recycled, moreover, it can be further oxidized to pterin-6-carboxylate. The oxidation can occur in both non-enzymatic and enzymatic ways (Noiriel et al., 2007a,b).

## Folate transport

Folate metabolism is known to function in several subcellular compartments. Several pieces of evidence suggested existence of interorganellar shuttling of folates and their precursors. First, THF synthesis includes import of precursors, pABA and pterin, from plastids and cytosol, respectively, into mitochondria. Second, although folates are synthesized in mitochondria, they are generously distributed throughout the plant cell, therefore, export of folates from the compartment must exist. Third, mutants compromised in folate biosynthesis can be rescued by exogenous folate application. Finally, folate transporters were found in other species.

Unlike pABA, which is known to penetrate intracellular membranes by diffusion (Quinlivan et al., 2003), pterins have to be transported into mitochondria in order to be used in folate synthesis. Although the ability of plants to take up and metabolize pterins is well known (Orsomando et al., 2006; Noiriel et al., 2007a,b), no plant pterin transporter has been identified to date. Only a transporter competent of shuttling biopterin into Leishmania cells has been described (Lemley et al., 1999).

Two transporters that facilitate folate transport into chloroplasts have been identified. Although being first identified as the closest mammalian mitochondrial folate transporter, AtFOL1 protein, encoded by At5g66380, was found to localize to the envelope of chloroplasts. It proved to function as a folate transporter in Chinese hamster ovary (CHO) cells and *E. coli* mutant background. Inactivation of At5g66380 affected neither the chloroplastic folate pool nor plant growth, suggesting existence of an alternative plastidial transporter (Bedhomme et al., 2005).

The alternative chloroplast-localized transporter encoded by At2g32040 gene rescues *E. coli* mutants unable to produce or transport folates. The Arabidopsis transporter was shown to shuttle 5-formyl-THF, folic acid and two antifolates, MTX and aminopterin. Interestingly, only monoglutamylated forms of folates could be transported (Klaus et al., 2005a). The Arabidosis genome contains 8 homologs of At2g32040 gene. However, none of the homologs proved to be functional in *E. coli* and Lieshmania folate and pterin transport mutant background (Eudes et al., 2010). The homologs were shown to lack some well-conserved residues, supposedly affecting folate binding ability. As only 5-formyl-THF transport was investigated in the study, it is still possible that the homologs transport other folate derivatives and might have diverse specificity for different folate species. Disruption of At2g32040 significantly enhanced total folate content of chloroplasts and lowered abundance of 5-formyl-THF, but did not affect plant growth and development, which reinforced the notion of functional redundancy between the two identified plastidial transporters.

A significant fraction of cellular folate pool localizes to vacuoles of the plant cell (Orsomando et al., 2005). Vacuolar folate transporters were identified in Arabidopsis and red beet. Vacuolar membrane localized multidrug resistance-associated protein (MRP) AtMRP1 in Arabidopsis and its counterpart from red beet were demonstrated to function as transporters of folate mono- and polyglutamates, as well as antifolates (Raichaudhuri et al., 2009). It was demonstrated that disruption of AtMRP1 causes elevated sensitivity to MTX, resulting from hampered sequestration of the toxic compound in vacuoles. The competence of the transporter to shuttle polyglutamylated forms of folates does not conform to the notion that folate transporters are specific for monoglutamylated forms of folates (Suh et al., 2001; Klaus et al., 2005a; Eudes et al., 2008b). Finding that MTX polyglutamates are transported from cytoplasm to lysosomes in mammalian cells (Barrueco and Sirotnak, 1991) suggested that transporters capable of shuttling polyglutamylated folates are not unique to plant vacuoles, moreover they can be conserved throughout evolution.

Transporters assisting the passage of folates into cytosol are known in mammals (Kamen et al., 1988). Mammalian cells were also reported to take up MTX using reduced folate carrier (RFC)-mediated transport (Goldman et al., 1968; Dixon et al., 1994). The notion that folates can be taken up by a plant cell is supported by numerous studies, demonstrating that exogenously supplied folate, 5-formyl-THF, can rescue mutants deficient in folate production (Mehrshahi et al., 2010; Meng et al., 2014; Reyes-Hernandez et al., 2014). Moreover, the notion is substantiated by the known ability of plant cells to take-up antifolate drugs such as MTX (Cella et al., 1983). Additionally, plant cells are capable of taking up pterins and using them in folate synthesis (Orsomando et al., 2006; Noiriel et al., 2007a,b).

Import of folates into mammalian mitochondria was found to be conducted by the inner membrane mitochondrial transport protein (MTP). MTP could reconstitute the accumulation of folates within the mitochondrial matrix in a CHO mutant cell line that was deficient in this process (McCarthy et al., 2004). Import of folates in mitochondria certainly exists in plants, since, as mentioned above, Arabidopsis mutants defective in folate biosynthesis can be rescued by application of 5-formyl-THF. To be metabolized, the folate derivative has to be converted to 5,10-methenyl-THF by the action of 5-formyl-THF cyclohydrolase (FTHFC), which localizes exclusively in mitochondria in plant cells (Roje et al., 2002b).

## Functions of folates

**Figure 4:**
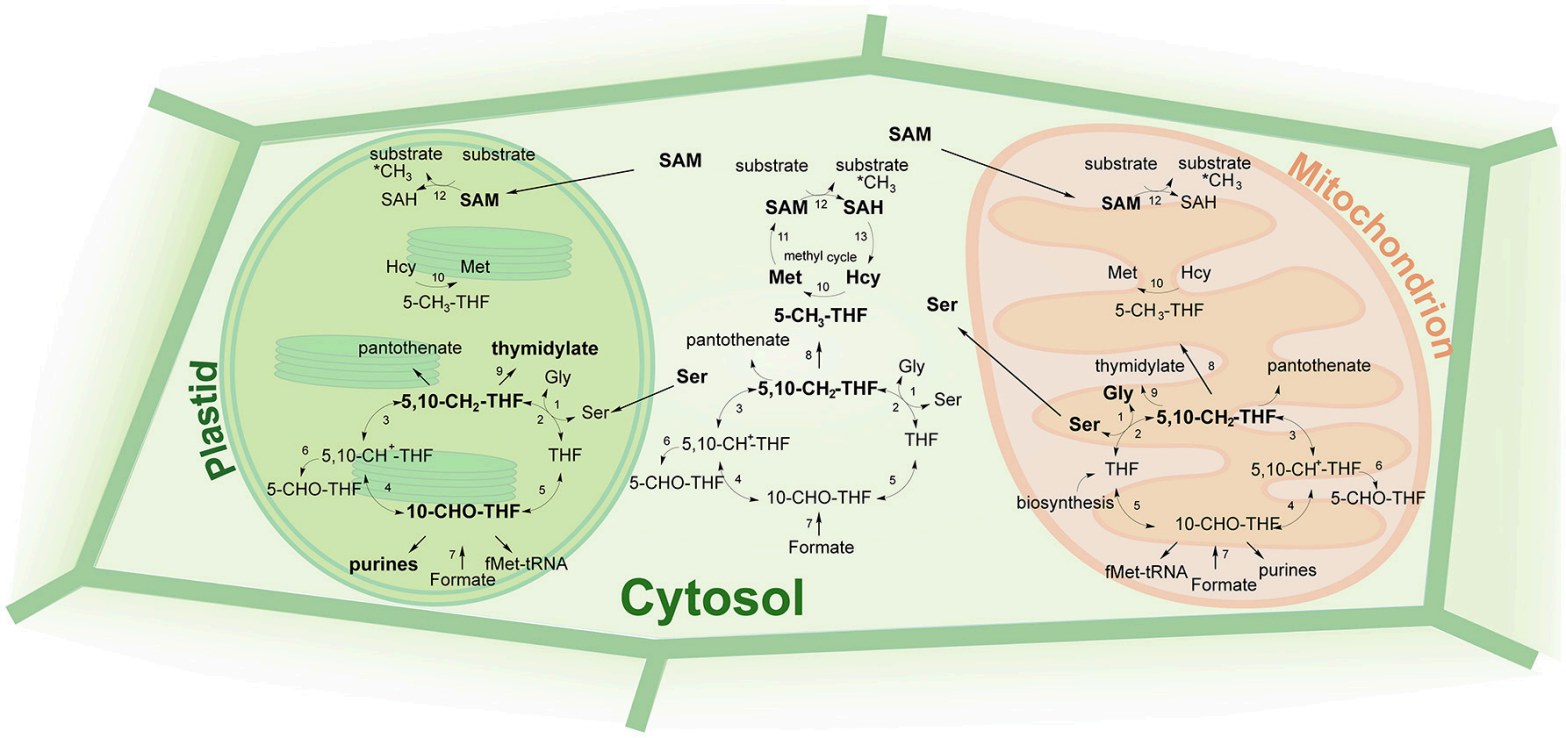
**Metabolic functions of folates**. THF, tetrahydrofolate; 5-CH_3_-THF, 5-methyltetrahydrofolate; 5,10-CH_2_-THF, 5,10-methylenetetrahydrofolate; 5,10-CH^+^-THF, 5,10-methenyltetrahydrofolate; 5-CHO-THF, 5-formyltetrahydrofolate; 10-CHO-THF, 10-formyltetrahydrofolate; SAM, S-adenosyl-methionine; SAH, S-adenosyl-homocysteine; Hcy, homocysteine; Met, methionine; Ser, serine; Gly, glycine. 1, serine hydroxymethyl transferase; 2, glycine decarboxylase complex; 3, 5,10-methylenetetrahydrofolate dehydrogenase; 4, 5,10-methenyltetrahydrofolate cyclohydrolase; 5, 10-formyltetrahydrofolate synthetase; 6, 5-formyltetrahydrofolate cyclohydrolase; 7, 10-formyltetrahydrofolte synthetase; 8, 5,10-methylenetetrahydrofolate reductase; 9, thymidylate synthase; 10, methionine synthase; 11, S-adenosylmethionine synthetase; 12, methyltransferase; 13, SAH hydrolase. Bolded reactions are specific for/ prevalent in a given subcellular compartment.

One of the most important cellular functions of folates is their implication in DNA synthesis. Thus, folate deficiency in animal tissues with rapidly dividing cells results in ineffective DNA synthesis (Kim, 2003; Kim et al., 2009). Lowered folate levels were also found associated with impaired nucleotide production in plants (Srivastava et al., 2011). Namely, two folate derivatives contribute to DNA synthesis: 10-formyl-THF and 5,10-methylene-THF (Figure 4). 10-formyl-THF is involved in the synthesis of purine nucleotides by donating its one-carbon unit to carbon atoms 2 and 8 of the purine ring (Rowe, 1984; Liu and Lynne Ward, 2010). This folate species is also used for the production of formyl-methionyl-tRNA (Staben and Rabinowitz, 1984; Schnorr et al., 1994) in the reaction catalyzed by methionyl-tRNA transformylase in mitochondria and chloroplasts (Cossins, 1987). 5,10-methylene-THF is used as a C1 donor by thymidylate synthase (TS) in the conversion of dUMP into dTMP that occurs mitochondria (Neuburger et al., 1996). The localization of bifunctional DHFR-TS proteins in carrot chloroplasts suggests that the conversion might take place in plastids as well (Luo et al., 1997). In the reaction 5,10-methylene-THF is converted into DHF that is subsequently reduced back to THF by DHFR enzymatic activity. Besides its role in thymidylate synthesis, 5,10-methylene-THF also serves as a C1 donor in the production of pantothenate (vitamin B5), a precursor of co-enzyme A, and in the conversion of glycine to serine, an important reaction during photorespiration in plants (Douce et al., 2001). Two subcellular compartments are potentially competent for nucleotide synthesis, namely mitochondria and plastids, as they both contain enzymes involved in the nucleotide production, such as TS, phosphoribosylglycinamide formyltransferase (GART) and phosphoribosylaminoimidazolecarboxamide formyltransferase (AICART). However, the flux of one-carbon units from folate metabolism toward nucleotide synthesis is assumed to occur primarily in plastids (Neuburger et al., 1996; Atkins et al., 1997; Luo et al., 1997). In agreement with this, C13 tracer studies demonstrated that the 5,10-methylene-THF pool in mitochondria predominantly shuttles between the glycine decarboxylase complex (GDC) and the mitochondrial serine hydroxymethyltransferase (mSHMT) in the interconversion of Gly and Ser, rather than participating in thymidylate synthesis (Prabhu et al., 1996; Mouillon et al., 1999).

5,10-methylene-THF can be reduced by MTHFR into 5-methyl-THF, that enters methyl cycle where the one-carbon group of 5-methyl-THF is utilized by methionine synthase in the conversion of homocysteine (Hcy) to methionine. This reaction was found to occur in plant mitochondria (Clandinin and Cossins, 1974), in chloroplasts (Shah and Cossins, 1970) and in cytosol (Eichel et al., 1995). Methionine is further used by SAM synthetase in the production of S-adenosyl-methionine (SAM), that is employed by methyltransferases for methylation of DNA, RNA, lipids, histones and other substrates (Crider et al., 2012). Upon donating its methyl group, SAM is converted to S-adenosyl-homocysteine (SAH). Being a potent inhibitor of MTHFR (Jencks and Mathews, 1987; Roje et al., 2002a), SAM regulates the flux of methyl groups from the folate pathway into methyl cycle. Thus, low SAM levels favor methionine production and vice versa (Crider et al., 2012). The one-carbon flux from folate metabolism toward methionine production is assumed to occur predominantly in the cytosol (Isegawa et al., 1993). The cytosol is also considered to be the only compartment capable of SAM production, since SAM synthetase is present exclusively therein (Hanson and Roje, 2001). It is therefore suggested that methylation reactions in plastids are supported by the transport of SAM from the cytosol (Ravanel et al., 2004) As SAH produced during methylation reactions is a potent inhibitor of methyltransferases (Moffatt and Weretilnyk, 2001), it has to be efficiently eliminated. The removal of SAH is implemented by SAH hydrolase that recycles of SAH into SAM and is assumed to be restricted to cytosol (Hanson and Roje, 2001). Export of SAH from chloroplasts was shown to be facilitated by a transporter that imports SAM from the cytosol (Ravanel et al., 2004). Besides being involved in methylation reactions, methionine can be used in protein synthesis.

Exclusively in plants, THF is involved in photorespiration that resides in three subcellular compartments: plastids, mitochondria and peroxisomes. During photorespiration, glycine is made from glycolate-2-P and is further converted to 5,10-methylene-THF and CO_2_ by GDC. 5,10-methylene-THF is then combined with a second glycine by mSHMT to make serine (Cossins, 2000; Douce et al., 2001). While serine serves as the major source of single-carbon groups through the reverse reaction of SHMT, folates play a role to transport one-carbon units. 5,10-methylene-THF establishes a central hub in one-carbon metabolism. It can be directly used for the production of thymidylate or pantothenate or can be converted to 5-methyl-THF that enters methyl cycle. Moreover, it can be used for 10-formyl-THF synthesis that is carried out through two sequential reactions catalyzed by a bifunctional methylene tetrahydrofolate dehydrogenase/methenyl tetrahydrofolate cyclohydrolase (MTHFD/MTHFC). 10-formyl-THF can be utilized in purine synthesis or in formyl-methionine-tRNA production. Finally, 10-formyl-THF is converted to THF, the unloaded folate form, by the action of 10-formyl-THF synthetase (FTHFS). The reloading of THF with a one-carbon unit is achieved through a reaction performed by SHMT where THF receives its one-carbon group from serine. Serine that is formed from glycine and 5,10-methylene-THF in mitochondria can be transported to the cytosol and plastids (Rébeillé et al., 1994), where it is converted into 5,10-methylene-THF by cytosolic and plastidial SHMT activities, respectively (Neuburger et al., 1996).

Another source of one-carbon units for folate metabolism is formate. Formate is converted to 10-formyl-THF in the reaction catalyzed by FTHFS. The presence of the enzyme in cytosol, plastids and mitochondria (Shingles et al., 1984; Kirk et al., 1994, 1995; Neuburger et al., 1996) suggests that formate indeed can be an alternative source of one-carbon groups for the folate metabolism.

## Distribution of folates

The total folate pool is mainly composed of five THF derivatives: 5-methyl-THF (45–65%), 5-formyl-THF and 10-formyl-THF (30–55%) and THF and 5,10-methylene-THF (10–15%) (Cossins, 2000; Loizeau et al., 2007; Van Wilder et al., 2009). Studies on folate composition in pea leaves demonstrated that folate derivatives are not equally distributed among subcellular compartments (Jabrin et al., 2003; Orsomando et al., 2005). Mitochondria, being the actual site of *de novo* folate synthesis, contain more folates than other subcellular compartments (40%), while cytosol holds 30% and plastids and vacuoles contain 20% and 10%, respectively, of the total cellular folate pool (Jabrin et al., 2003; Orsomando et al., 2005). One may expect that folate species are distributed within a cell according to their function and the metabolic role of the compartment they reside in.

Thus, mitochondrial folate pool is dominated by THF and its formyl derivatives (Orsomando et al., 2005). THF is synthesized in mitochondria and further either directly transported to other subcellular compartments, or first loaded with one-carbon units and then transported from mitochondria 5-formyl-THF, although is not being used as a one-carbon donor, represents 50% of the formyl pool of mitochondria (Orsomando et al., 2005). It presumably serves as a storage folate form, which can be converted to folate derivatives that can be used in one-carbon transfer reactions. Another possible function of 5-formyl-THF in mitochondria is the regulation of folate-dependent enzymes. For instance, it is known to inhibit activity of mitochondrial SHMT, thereby tuning the rate of photorespiration (Roje et al., 2002b; Goyer et al., 2005).

The prevailing folate derivative in the cytosol is 5-methyl-THF (Chen et al., 1997) which is readily used for methionine synthesis coupled with SAM production to support methylation reactions. The plastidial folate pool is dominated by 5-methyl-THF and 10-formyl-THF (Orsomando et al., 2005) which can be employed in methionine production and purine synthesis, respectively. Both processes were shown to occur in plastids (Ravanel et al., 2004; Zrenner et al., 2006).

The length of the polyglutamate tail is another factor known to affect distribution of folate species within a cell. Polyglutamate tails ensure retention of folates in subcellular compartments (Appling, 1991). Moreover polyglutamylated folate forms are preferred by folate-dependent enzymes and folate transporters over their monoglutamylated counterparts (Rébeillé et al., 1994). Interestingly, when polyglutamylated folates are associated with folate-dependent enzymes they are less susceptible to oxidative degradation than when they are free in solution (Rébeillé et al., 1994). Most of cellular folates are polyglutamates with 4–6 glutamate residues (Besson et al., 1993; Cossins, 2000).

Besides being unequally distributed between subcellular compartments, folates vary in their abundance in different tissues. Numerous studies pointed out that folate biosynthesis is most active in dividing tissues, such as root tips of pea and maize (Cox et al., 1999; Jabrin et al., 2003) or actively growing Arabidopsis cell suspension cultures (Loizeau et al., 2007, 2008). High expression of folate biosynthesis genes was found in meristems, expanding cotyledons and developing carrot embryos (Albani et al., 2005). High transcript abundance of folate biosynthesis genes and elevated folate levels were also detected in pea embryos (Jabrin et al., 2003). The elevated level of folates in these tissues might reflect a high demand for one-carbon units for the nucleotide synthesis during the S-phase, as the genome is doubled. In agreement with this, the expression of ribonucleotide reductase, the enzyme involved in nucleotide synthesis, is also found to be induced at the G1/S transition (Chabouté et al., 1998). Thus, it is very likely that genes of the folate biosynthesis pathway are also regulated in a cell-cycle dependent manner.

## Quantitative analysis of folates in plant samples, a prerequisite in biofortification programs

In order to control the success of biofortification efforts, appropriate analytical methods are required to determine folate content in engineered plants. In experiments with genetically modified plants, analysts are often provided with only small amounts of sample material. Further challenges in folate analysis include the distinction between different folate species, their instability and the complexity of the sample matrices (De Brouwer et al., 2008).

Several methods for different food matrices such as fruit, juice, vegetables, potatoes, eggs, milk, meat and cereals have been published (Pfeiffer et al., 1997; Rader et al., 1998; Konings et al., 2001; Ndaw et al., 2001; Freisleben et al., 2003a,b; Rychlik, 2004; De Brouwer et al., 2008, 2010; Van Daele et al., 2014). During sample preparation, it is crucial to consider conditions that support the stabilization of folates, since folates are light-sensitive as well as sensitive to oxidants, reducers, acids and bases (Fitzpatrick et al., 2012). Interconversion between different folate species is also possible, especially for formyl folates (Jägerstad and Jastrebova, 2013). Therefore, samples should generally be stored at −80°C, all sample manipulations should be carried out under subdued light conditions and antioxidants should be employed in sample preparation (Van Daele et al., 2014; Strandler et al., 2015). Ascorbic acid is commonly used as an antioxidant. However, ascorbic acid can form formaldehyde when heated, which can in turn cause interconversion of folates. For this reason, thiols such as dithiothreitol (DTT) are also added to the extraction buffer to capture formaldehyde (De Brouwer et al., 2008; Strandler et al., 2015). Lyophilization of samples can be used to express the folate content per dry-weight but does not have a positive effect on folate stabilization (Stea et al., 2007; Goyer and Sweek, 2011; Van Daele et al., 2014). For an extensive review of factors affecting the stability and interconversion of folates, the publication by Strandler et al. is recommended (Strandler et al., 2015).

For starchy matrices, such as potato and rice, and for milk, the use of a tri-enzyme treatment consisting of amylase, protease and conjugase has proven beneficial (Martin et al., 1990; De Brouwer et al., 2008; Van Daele et al., 2014). Amylase and protease are used to degrade starchy and protein components of the sample matrix, respectively, while conjugase cleaves the folate polyglutamate tail to produce mono- or diglutamates, depending on the source of conjugase (Strandler et al., 2015). Alternatively, the addition of conjugase can be skipped if only free monoglutamates are to be determined. For the analysis of folates in potatoes, tri-enzyme treatment did not enhance extraction efficiency but did facilitate sample handling through matrix degradation; hence it may decrease potential variability when different tuber sections are analyzed (Van Daele et al., 2014). In contrast, tri-enzyme treatment is necessary in the analysis of rice for optimal folate extraction (De Brouwer et al., 2008). Boiling steps are also used in the preparation of starchy samples to soften the sample matrix and to reduce enzymatic and oxidative degradation (Van Daele et al., 2014). For leafy matrices such as *Arabidopsis thaliana*, enzymatic treatment, except with conjugase where desired, is not required.

Extraction after tri-enzyme treatment can be followed by simple ultracentrifugation (De Brouwer et al., 2008, 2010; Van Daele et al., 2014). More laborious and complex sample preparation protocols may involve solid phase extraction (SPE) or affinity-based purification involving folate-binding protein (Wilson and Horne, 1984; Konings et al., 2001; Hyun and Tamura, 2005).

**Figure 5:**
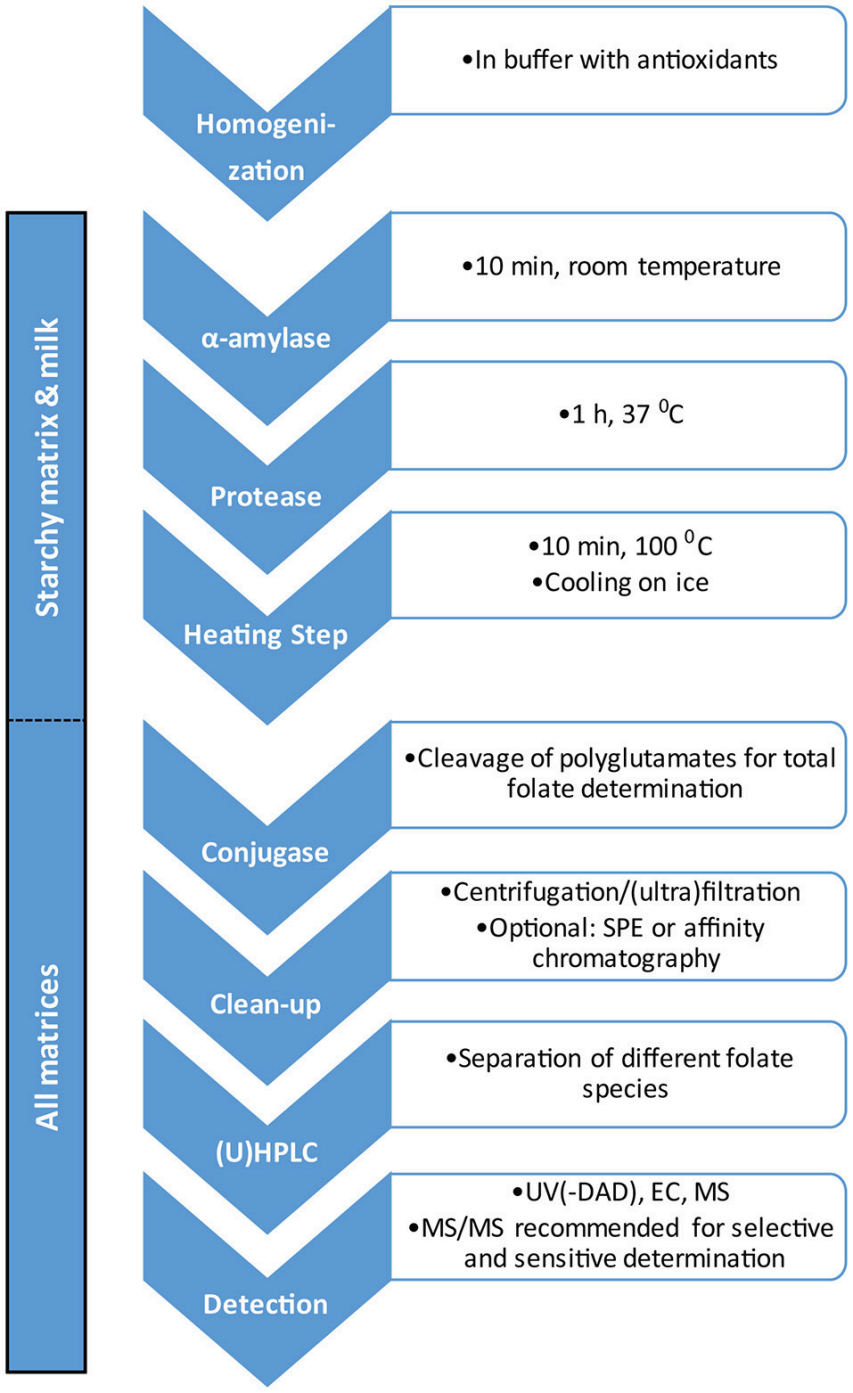
**Flow chart for determination of folates in plant material**.

Different techniques for the determination of folates in sample extracts have been reported. Historically, microbiological assays were the method of choice. However, these assays are time-consuming and cannot distinguish between different folate species (Strandler et al., 2015). In order to achieve this distinction, chromatographic methods are required. While a number of different detectors such as UV(-DAD), fluorescence, electrochemical and mass spectrometric detectors have been reported in conjunction with high performance liquid chromatography (HPLC) (Pfeiffer et al., 1997; Bagley and Selhub, 2000; Ndaw et al., 2001; Rychlik and Freisleben, 2002; Doherty and Beecher, 2003; Freisleben et al., 2003a; Zhang et al., 2003; Nelson et al., 2004; Fazili et al., 2005; Garratt et al., 2005; Zhang et al., 2005; De Brouwer et al., 2007; Patring and Jastrebova, 2007; Gutzeit et al., 2008), (Ultra-) HPLC coupled to tandem mass spectrometry [(U)HPLC-MS/MS] has become the *de facto* standard for the sensitive and selective determination of folates (De Brouwer et al., 2010). UHPLC-MS/MS has also been employed for the two-dimensional characterization of folate content in potato tubers (Van Daele et al., 2014). For quantitative methods using mass spectrometric detection, the use of isotope-labeled internal standards is essential in order to compensate for both analyte loss during sample preparation and for matrix effects (De Brouwer et al., 2008, 2010; Van Daele et al., 2014). Additionally, the use of a stable isotope dilution assay for the determination of folates has been reported (Rychlik, 2004; Ringling and Rychlik, 2013). A flow chart outlining the general analytical steps for the determination of folates in plant material is shown in Figure 5.

## Biofortification

**Figure 6:**
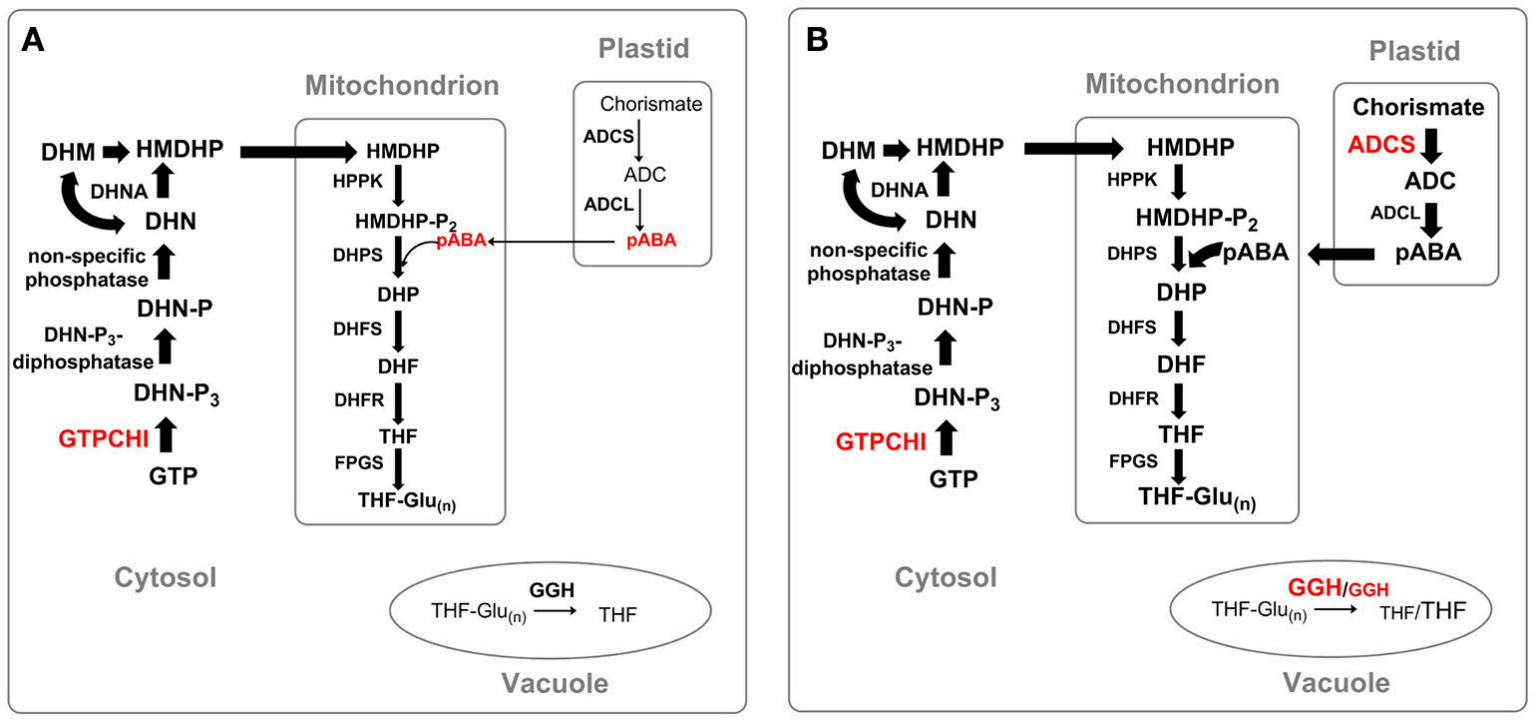
**Strategies of enhancement of folate level in plants. (A)** Manipulation of GTPCHI expression, **(B)** manipulation of GTPCHI, ADCS and GGH expression. Thickness of arrows indicates the efficiency of metabolic flux through the pathway. Precursors: GTP, guanosine triphosphate; DHN-P_3_, dihydroneopterin triphosphate; DHN-P, dihydroneopterin monophosphate; DHN, dihydroneopterin; HMDHP, 6-hydroxymethyldihydropterin; HMDHP-P_2_, 6-hydroxymethyldihydropterin pyrophosphate; DHP, dihydropteroate; DHF, dihydrofolate; THF, tetrahydrofolate; THF-Glu_(n)_, tetrahydrofolate polyglutamate; ADC, aminodeoxychorismate; pABA, para-aminobenzoic acid. Enzymes: GTPCHI, GTP cyclohydrolase I; DHN-P_3_-diphosphatase, dihydroneopterin triphosphate pyrophosphatase; DHNA, dihydroneopterine aldolase; HPPK, HMDHP pyrophosphokinase; DHPS, dihydropteroate synthase; DHFR, dihydrofolate reductase; FPGS, folylpolyglutamate synthetase; ADCS, aminodeoxychorismate synthase; ADCL, aminodeoxychorismate lyase; GGH, gamma-glutamyl hydrolase.

A large part of the world's population is still suffering a severe deficiency of folate supply. To solve this problem, folate supplementation in a form of folic acid pills was launched. Folic acid undergoes two rounds of reduction by DHFR to be converted to the biologically active form, THF. Albeit being relatively simple and highly efficient, the approach turned out to be rather prohibitive in poor regions of the world, as the pills often don't reach the target populations. In addition to supplementation, industrial fortification of food products (e.g., flour) can be a possible approach to reduce folate deficiency. However, the necessary infrastructure is often lacking in developing countries, especially in rural areas. An alternative approach to fight folate deficiency is the enhancement of folate levels in staple crops. Both molecular breeding technology and metabolic engineering can be used to create crops with enhanced micronutrient content, each with their advantages and shortcomings (Blancquaert et al., 2017; Strobbe and Van Der Straeten, 2017). However, the first one is dependent on the natural variation in germplasm of the crop of interest. Natural variation of folate content in rice (Abilgos Ramos, 2010; Blancquaert et al., 2010) and in potato (Goyer and Navarre, 2007) was found to be relatively low, hampering successful breeding, keeping target levels in processed food in mind. In several studies on the enhancement of folate levels in plants, GTPCHI or ADCS (or both), were overexpressed (Figure 6). The approach aims at the enhancement of the metabolic flux through the biosynthetic pathway by increasing the supply of the two folate precursors—pterin and pABA. Overexpression of a codon-optimized native GTPCHI in lettuce and *E. coli* GTPCHI in corn plants alone resulted in only 2.1–8.5-fold and 2-fold, respectively, increase in the folate level (Naqvi et al., 2009; Nunes et al., 2009). The limited success of the two attempts was ascribed to the depletion of the pABA pool. In agreement with this notion, a severe depletion of pABA was observed in tomato plants overexpressing GTPCHI (Díaz de la Garza et al., 2004). In order to overcome this obstacle, a simultaneous overexpression of GTPCHI and ADCS was ventured. The approach successfully increased total folate content up to 25 and 100 times in studies on tomato and rice, respectively (de La Garza et al., 2007; Storozhenko et al., 2007a). Interestingly, the overexpression of ADCS alone lead to a decrease in the total folate content (Storozhenko et al., 2007a). It is possible that, being an inhibitor of FPGS (Storozhenko et al., 2007a), pABA impedes folate polyglutamylation, thus affecting folate stability and consequently its abundance. A study on rice demonstrated that overexpression of HPPK-DHPS, a gene coding for the first two enzymes of the mitochondrial part of the biosynthetic path, results in a slight increase of the folate level (Gillies et al., 2008). This result holds promise for possibility of a further increase in folate level by a combined overexpression of GTPCHI, ADCS and HPPK-DHPS genes.

Manipulation of the folate polyglutamylation status by altering GGH expression was also demonstrated to affect folate content. A study on tomato plants, reported that a reduction of GGH expression by means of RNA interference, lead to an increase in polyglutamate tail and an increase of the folate pool by 37%, whereas the overexpression of GGH slashed folate content by 40% (Akhtar et al., 2010).

Due to the inherent instability of folate molecules, the total folate pool is prone to degradation upon fruit ripening and storage. A recent study demonstrated that the expression of animal folate binding proteins increases stability of folates upon storage (Blancquaert et al., 2015), probably because folates are less susceptible to oxidative damage when associated with proteins (Rébeillé et al., 1994).

As future perspective for the enhancement of abundance and stability of the folate pool in staple crops a combination of several approaches should be ventured. It also should be taken into account that biofortification strategies applied may not be equally efficient for all staple crop species. Thus, overexpression of both ADCS and GTPCHI substantially increased folate level in tomato fruit (de La Garza et al., 2007) and rice grains (Storozhenko et al., 2007a), but did not affect that of Arabidopsis and potato tubers (Blancquaert et al., 2013a). Another example of possible species-specific regulation of folate synthesis is shown by two studies on enhancement of pABA pool, where while the enhancement of pABA pool did not affect total folate level in tomato plants (de La Garza et al., 2007), the increase of pABA level in rice resulted in a decrease of folate abundance (Storozhenko et al., 2007a). Therefore, in order to develop a successful biofortification strategy, one should consider possible differences in the regulation of folate biosynthesis of a given plant species.

## Roles of folates in plant physiology

### Folates and genome stability

Since the genome encodes every developmental and metabolic process in all living organisms, maintaining of its integrity is of pivotal importance. Folates play a tremendously important role in maintaining genome stability (Fenech, 2001). Three folate species are involved in this process: 5,10-methylene-THF, 10-formyl-THF and 5-methyl-THF. As was mentioned above, 5,10-methylene-THF is utilized in the conversion of dUMP into dTMP catalyzed by thymidylate synthase (TS). An insufficient conversion rate of dUMP to dTMP leads to a depletion of dTMP pool causing misincorporation of dUMP in DNA which ultimately affects genome stability. 10-formyl-THF is used for purine and formyl-methionyl-tRNA synthesis, thus being involved in nucleotide pool maintenance and translation. 5-methyl-THF provides its methyl group for the production of methionine that is further used in the formation of the most important methyl group donor for methylation reactions—SAM. Upon donating its methyl group, SAM is converted into S-adenosyl-homocysteine (SAH). Since SAH is a potent inhibitor of methytransferases, its removal is essential for the efficiency of methylation reactions (Molloy, 2012). The importance of methylation reactions in the maintenance and regulation of genomes (epigenetic control) receives increasing attention. As folates play a role of methyl group donors, a positive correlation between the folate level and DNA methylation status seems rather intuitive. However, numerous studies demonstrate that this scenario is not always realized. As was demonstrated in animal systems, folate deficiency can lead to global DNA hypomethylation (Balaghi and Wagner, 1993), to global DNA hypomethylation with hypermethylation at specific genome regions (Jhaveri et al., 2001; Pogribny and James, 2002), or to global DNA hypermethylation (Song et al., 2000; Sohn et al., 2003). Unlike animal systems, the role of folate metabolism in plant epigenome maintenance only starts to draw attention of researchers. So far, only examples of positive correlation between the folate level and DNA methylation have been reported. Thus, Arabidopsis plants depleted in folates by the treatment with the folate biosynthesis inhibitor sulfamethazine were shown to have reduced levels of DNA methylation, histone H3K9 dimethylation and to exhibit release of epigenetic silencing (Zhang et al., 2012). The same study demonstrated a release of transcriptional gene silencing in plants treated with methotrexate (MTX). Furthermore, disruption of the plastidial isoform of FPGS in Arabidopsis was reported to result in reduced DNA methylation and release of chromatin silencing at a genome-wide scale (Zhou et al., 2013).

Modification of histones establishes another important aspect of epigenome. Like other histone marks, methylation of histones can cause both transcriptional activation or silencing (Spencer et al., 1997; Xu et al., 1999; Lee et al., 2006). Recent studies demonstrate that transcription factors can also be subjected to methylation, that can alter their DNA binding and protein binding efficiency (Salbaum and Kappen, 2011).

Since methylation of DNA, histones and transcription factors is of paramount importance in regulation of gene expression, the involvement of folate metabolism in methylation reactions links it to virtually every process in a cell.

### Folates in plant development

Due to their crucial role in methylation reactions and in the overall cellular metabolism, folates are absolutely essential for development. The importance of folates for plant development was demonstrated by several studies reporting a severe repression of growth caused by inhibition of folate biosynthesis by application of antifolates (Crosti, 1981; Camara et al., 2012; Navarrete et al., 2013).

Several lines of evidence indicate that folates are indispensable during embryogenesis. Thus, *fpgs1/fpgs2* and *dhfs* (*gla1*) mutants compromised in folate biosynthesis are embryo lethal (Ishikawa et al., 2003; Mehrshahi et al., 2010). Moreover, the folate level in pea embryos was shown to be high (Gambonnet et al., 2001; Jabrin et al., 2003) and accompanied by high expression of genes involved in folate biosynthesis (Jabrin et al., 2003). This evidence suggested that plant embryos are autonomous for folate synthesis. Application of inhibitors of folate synthesis indicated that embryos not only produce folates necessary for their own proper development but also procure folates that are used during early postembryonic development (Gambonnet et al., 2001). As was shown in the experiment using antifolates, folates supply accumulated during embryonic development is sufficient for supporting cell division only during early post-germination growth and it soon becomes a limiting factor (Gambonnet et al., 2001). Therefore, folate biosynthesis is assumed to resume shortly after germination. During organ differentiation folate synthesis was found to be prevalent in highly dividing tissues, such as pea and maize root tips, maize kernels as well as in actively growing cell suspension cells of carrot (Cox et al., 1999; Jabrin et al., 2003; Albani et al., 2005).

The crucial role of folates in root development was demonstrated by recent studies using Arabidopsis plants mutant for the plastidial isoform of FPGS (AtDFB). While mutants for other FPGS isoforms did not exhibit obvious primary root defects, *atdfb* seedlings had a severely shortened primary root (Srivastava et al., 2011; Reyes-Hernandez et al., 2014). This pointed to specificity of the plastidial isoform in maintaining C1 metabolism during root development. Although the total folate content in *atdfb* seedlings was not altered, folate species distribution was disturbed. Mutant seedlings were also shown to be depleted in amino acids and nucleotides. The lowered nucleotide content was in agreement with significantly decreased level of 10-formyl-THF which is used for the synthesis of purines. Furthermore, the mutants exhibited a lowered methionine level (Srivastava et al., 2011) and decreased abundance of 5-methyl-THF which donates its methyl group to homocysteine in methionine synthesis (Ravanel et al., 2004; Rébeillé et al., 2006). Apparently, the plastidial isoform of FPGS is indispensable for syntheses of purine and methionine that are known to take place in plastids.

The primary root defect of *atdfb* plants was attributed to erroneous regulation of the root determinacy-to-indeterminacy switch (IDS) which is accompanied by the activation of the quiescent center (QC) and subsequent consumption of the root apical meristem (RAM). The activation of the QC was demonstrated to be dependent neither on auxin gradients nor on SHORTROOT/SCARECROW (SHR/SCR) and PLETHORA (PLT) pathways of RAM indeterminacy (Reyes-Hernandez et al., 2014). Taking into account the pivotal role of auxin in root development, the IDS independence of auxin gradients demonstrated by the mutants seemed quite striking. Nevertheless, the result is supported by a study showing a similar lack of a direct link with auxin, reporting that RAM exhaustion and root determinacy of triple mutant of GRAS TF HAIRY MERISTEM 1,2,3 is independent of auxin gradients (Engstrom et al., 2011).

Some pieces of evidence suggest that folates implement an important role in hypocotyl development. Thus, a crosstalk between folate and sucrose was shown to influence auxin signaling through a subset of IAA/ARFs to impact/regulate hypocotyl elongation (Stokes et al., 2013). Moreover, a crosstalk between folates and sucrose was previously observed in a study where seedling lethality of fpgs2 fpgs3 double mutants could be partially rescued by supplementation with sucrose (Mehrshahi et al., 2010). Another example of the involvement of folate metabolism in hypocotyl elongation is presented in a study revealing compromised hypocotyl elongation in *atdfb* mutants during skotomorphogenesis (Meng et al., 2014).

### Folates and light

Folate synthesis and accumulation were found to be elevated in leaves upon exposure to light (Jabrin et al., 2003). This finding pointed out a high demand for folates and thus one-carbon transfer reactions in metabolism of leaf photosynthetic tissues. This notion was corroborated by the study demonstrating a decrease in both total folate abundance and chlorophyll level in pea leaves upon application of an antifolate drug MTX (Van Wilder et al., 2009). The folate role in the methyl cycle was suggested to be accountable for the link between chlorophyll and folate levels. Thus, SAM, which synthesis involves 5-methyl-THF, is used by methytransferases that methylate numerous substrates, including a precursor of chlorophyll (Von Wettstein et al., 1995; Suzuki et al., 1997). Moreover, some chloroplastic enzymes such as Rubisco are also methylated (Black et al., 1987). Therefore, it is assumed that a high level of folates in leaves is needed for the proper functioning of the photosynthetic apparatus. Beside their role in chlorophyll synthesis, folates establish an additional link with plant light response. Folate molecules were shown to bind cryptochromes cry1 and cry2 and act as light-harvesting antenna pigment, thereby participating in blue-light perception (Hoang et al., 2008). It has been demonstrated that in purified preparations of CRY-DASH-type cryptochromes of *Vibrio cholera* folate as a light-sensing antenna pigment is involved in energy transfer to flavin which is the second chromophore that binds to cryptochromes (Saxena et al., 2005). It is possible that a similar mechanism of blue-light perception operates in plants. The enhancement of folate biosynthesis upon light exposure could also be attributed to the role of folates in the Gly-to-Ser transition during photorespiration. Upon light exposure the expression of HPPK-DHPS was demonstrated to follow the accumulation of transcripts of GDC and SHMT that link folate metabolism to photorespiration (Jabrin et al., 2003).

### Folates and nitrogen reserves

Nitrogen is an essential macronutrient and one of the major limiting factors for plant growth (Diaz et al., 2006). Insufficient nitrogen supplementation results in perturbations of plant metabolism and severe growth defects (Martin et al., 2002; Diaz et al., 2008; Lemaître et al., 2008). Thus, Arabidopsis seedlings mutant for either plastidial or mitochondrial FPGS demonstrate erroneous folate species distribution and display a disturbed nitrogen metabolism within a specific developmental window (Jiang et al., 2013; Meng et al., 2014). Mutants for the plastidial form of FPGS were shown to be defective in seed reserves exhibiting a defective C and N partitioning capacity (Meng et al., 2014). Plants lacking the mitochondrial FPGS isoform demonstrated root shortening upon nitrogen-limited conditions (Jiang et al., 2013). Both mutants exhibited a typical phenotype of plants under low-nitrogen stress, showing lowered soluble protein content, decreased free amino acids abundance, low nitrate content and decreased abundance of nitrogen storage amino acids and accumulation of ${\text{NH}}_{4}^{+}$. Since non-photorespiratory conditions could partially rescue the phenotype of plants lacking mitochondrial FPGS under N-limited conditions, the growth defects were assumed to be in part caused by impaired photorespiration (Jiang et al., 2013).

### Folates in stress response

With a future of climatic instability, production of stress resistant crops will become the major challenge in the near future. Deeper understanding of metabolic responses and signaling would help to identify best candidates for genetic manipulations for enhancement of crop resistance and productivity. Since plant stress responses include adjustment of a galore of metabolic processes and folates take part in a good deal of them, a tight and elaborate regulation of folate biosynthesis and metabolism in response to various stress conditions can be expected. However, the role of folates in plant stress response and resistance was largely overlooked. Studies conducting transcriptome and metabolome analyses provided evidence that folate metabolism is dramatically and differentially affected by various stress conditions. On the one hand, a study on Arabidopsis suspension cell cultures reported an up-regulation of genes involved in folate biosynthesis and consumption in response to oxidative stress imposed by application of menadione (Baxter et al., 2007). On the other hand, a global protein expression study investigating the proteomic response of rice plants to cold stress, demonstrated a down-regulation of folate biosynthesis (Neilson et al., 2011). A decrease of folate biosynthesis related genes was also observed in plants under salt stress (Storozhenko et al., 2007b).

A link between folate depletion and oxidative stress in animals was demonstrated by numerous studies (Cano et al., 2001; Huang et al., 2001; Ho et al., 2003; Dhitavat et al., 2005). In general, oxidative stress is caused by accumulation of reactive oxygen species (ROS) that include free radicals such as superoxide anion (${\text{O}}_{2}^{-}$), hydroxyl radical (HO°), as well as non-radical molecules including hydrogen peroxide (H_2_O_2_) and singlet oxygen. In plants, ROS are largely formed by the leakage of electrons on O_2_ from the electron transport activities of chloroplast, mitochondria and plasma membranes or as byproducts of various processes from different compartments (Foyer and Harbinson, 1994; del Río et al., 2006; Blokhina and Fagerstedt, 2010; Heyno et al., 2011). High levels of ROS are very harmful to an organism and therefore need to be neutralized by the cellular antioxidant system. The antioxidant system comprises enzymatic and non-enzymatic antioxidants. Superoxide dismutase (SOD), catalase (CAT), thioredoxin (TXN), and guaiacol peroxidase (GPX) establish enzymatic defense against ROS (Noctor and Foyer, 1998) while glutathione (GSH) and ascorbate (AsA), the main components of Asada-Halliway pathway, are the major cellular redox buffers that constitute the non-enzymatic ROS detoxifying system.

Besides being used in reductive biosynthetic processes involving those of nucleotide and fatty acid production, NADPH is used by the glutathione-ascorbate ROS detoxifying pathway as a reducing agent for glutathione recycling. The cofactor is also employed by the enzymatic antioxidant thioredoxin. The major sources of NADPH production in plants are the photosynthetic electron transfer chain in plastids (Kramer and Evans, 2011) and the oxidative pentose phosphate pathway, that takes place in the cytosol and in plastids (Kruger and von Schaewen, 2003). Mitochondria, as the actual (major) site of folate biosynthesis (Neuburger et al., 1996), contribute to a lesser extent to NADPH accumulation. Reduction of NADP^+^ is attributed to a number of mitochondrial enzymes [isocitrate dehydrogenase (EC 1.1.1.42), malic enzyme (EC 1.1.1.39), delta-pyrroline-5- carboxylate dehydrogenase (EC 1.5.1.12), glutamate dehydrogenase (EC 1.4.1.3) and methylenetetrahydrofolate dehydrogenase (EC 1.5.1.5) (Rasmusson and Møller, 1990; Møller and Rasmusson, 1998)]. The actual contribution of each of the mentioned sources to the total NADPH production in plants remains unclear to date.

**Figure 7:**
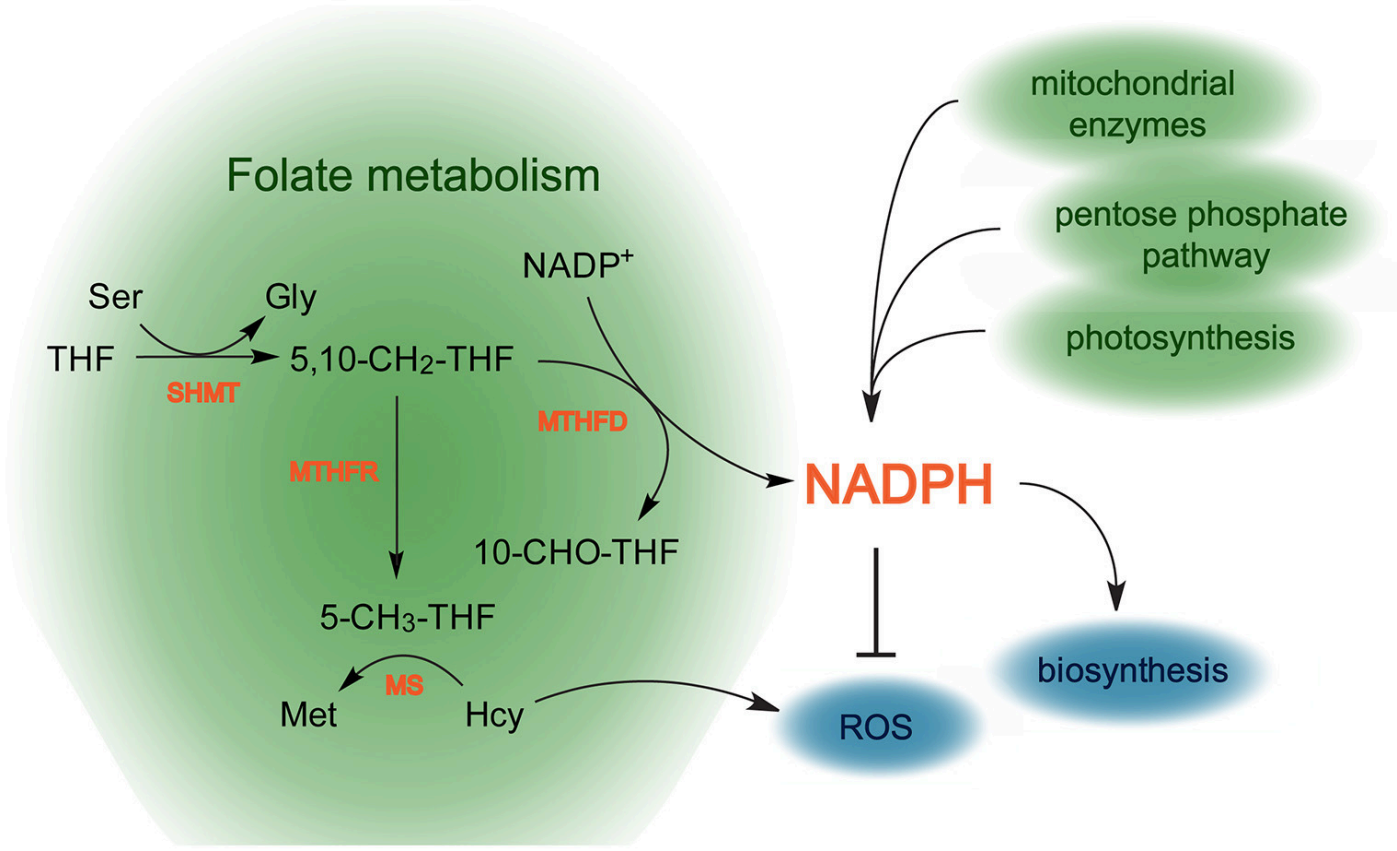
**Scheme reflecting the role of folate metabolism in redox homeostasis in plants**. The folate pathway possibly contributes to production of NADPH that is used to detoxify ROS. Additionally, 5-CH_3_-THF takes part in the conversion of Hcy into Met, thereby preventing ROS production. Green areas represent pathways contributing to NADPH production, blue areas show pathways consuming NADPH, biosynthetic processes and ROS removal. THF, tetrahydrofolate; 5-CH_3_-THF, 5-methyltetrahydrofolate; 5,10-CH_2_-THF, 5,10-methylenetetrahydrofolate; 10-CHO-THF, 10-formyltetrahydrofolate.

A recent NADP^+^ labeling study demonstrated that folate metabolism contributes to the total NADPH production in animals. The reaction that results in NADPH production converts 5,10-methylene-THF into 10-formyl-THF and is performed by MTHFD (Fan et al., 2014) (Figure 7). The study demonstrated that metastatic tumor cells exhibit an elevation in the production of NADPH, associated with increased activity of the folate pathway. This suggests that tumor cells evolved an adaptation to buffer oxidative stress by activating the folate pathway (Fan et al., 2014).

The relationship of folate metabolism with oxidative stress does not seem to be restricted by the production of NADPH, though. Several studies suggest that folate pathway might be linked with ROS metabolism through homocysteine. Thus, an increase of homocysteine level caused by low folate intake in humans (Jacob et al., 1994; Bostom et al., 1996; Shimakawa et al., 1997) was found to exert toxicity on endothelial cells by increasing H_2_O_2_ production (Starkebaum and Harlan, 1986), affecting antioxidant defense systems (Blundell et al., 1996). Being in agreement with the data, another study demonstrated that culturing embryonic cortical neurons and differentiated SH-SY-5Y human neuroblastoma cells in folate-free medium induced neurodegenerative changes that were accompanied by increased ROS level resulted from elevation of homocysteine abundance (Ho et al., 2003).

Since folate metabolism is an essential part of basic cellular metabolism, one can expect a great conservation in its functioning, roles and regulation throughout evolution. Therefore, it is possible that the role of the folate pathway in ROS metabolism revealed in animal systems can be also found in plants.

Several lines of evidence associate folates with plant innate immunity. Thus, expression of defense-related genes is induced in rice seeds with elevated folate level (Blancquaert et al., 2013b). Furthermore, application of pABA induced systemic acquired resistance (SAR) against artificially infiltrated *Xanthomonas axonopodis* and naturally occurring tobacco mosaic virus in pepper seedlings (Song et al., 2013). The importance of folate metabolism in biotic stress resistance was also demonstrated in a study assessing global gene expression patterns in response to the fungal pathogen *Fusarium pseudograminearum* (Powell et al., 2016). Finally, folate precursor DHN and folic acid induce local and systemic SA-mediated defense in Arabidopsis (Wittek et al., 2015). Altogether, these findings suggest that folate metabolism might have an important role in plant biotic stress response.

## Conclusions

Until recently, plant folate field was dominated by biofortification studies. Although a significant amount of knowledge regarding folate biochemistry and biosynthesis in various plant species has been accumulated, roles of folates in plant physiology were largely overlooked. The implication of folates in many essential processes such as synthesis of DNA and amino acids, methylation reactions suggests that these compounds might affect virtually every process in plants. Recent findings provided compelling evidence for involvement of folates in various aspects of plant physiology such as development, light response, nitrogen and carbon metabolism and stress response. Perturbations to any of the listed aspects lead to impaired plant growth and loss of productivity and ultimately affects agricultural value of crops. Although very scarce, the evidence on the role of folates in stress response holds promise for improvement of plant performance in unfavorable conditions. Study of physiological roles of folates would not only deepen our understanding of functions of these compounds in plants but also has a potential to fill gaps in studies of various processes in a plant cell.

## Author contributions

VG conducted the literature search, drafted the manuscript and prepared figures. LA drafted the part on folate analysis and prepared Figure 5. FR, CS and DV helped in writing the final manuscript.

### Conflict of interest statement

The authors declare that the research was conducted in the absence of any commercial or financial relationships that could be construed as a potential conflict of interest.

## References

[B1] Abilgos Ramos R. (2010). Folate Profiling in Wild and Transgenic Rice. Nottingham: University of Nottingham.

[B2] Akhtar T. A. McQuinn R. P. Naponelli V. Gregory J. F. III Giovannoni J. J. Hanson A. D. (2008). Tomato γ-glutamylhydrolases: expression, characterization, and evidence for heterodimer formation. Plant Physiol. 148, 775–785. 10.1104/pp.108.124479 18757550 PMC2556829

[B3] Akhtar T. A. Orsomando G. Mehrshahi P. Lara-Núñez A. Bennett M. J. Gregory J. F. III. (2010). A central role for gamma-glutamyl hydrolases in plant folate homeostasis. Plant J. 64, 256–266. 10.1111/j.1365-313X.2010.04330.x 21070406

[B4] Albani D. Giorgetti L. Pitto L. Luo M. Cantoni R. M. (2005). Proliferation-dependent pattern of expression of a dihydrofolate reductase-thymidylate synthase gene from *Daucus carota*. Eur. J. Histochem. 49, 107. 15967738

[B5] Appling D. R. (1991). Compartmentation of folate-mediated one-carbon metabolism in eukaryotes. FASEB J. 5, 2645–2651. 1916088 10.1096/fasebj.5.12.1916088

[B6] Arcot J. Shrestha A. (2005). Folate: methods of analysis. Trends Food Sci. Technol. 16, 253–266. 10.1016/j.tifs.2005.03.013

[B7] Atkins C. A. Smith P. Storer P. J. (1997). Reexamination of the intracellular localization of *de novo* purine synthesis in cowpea nodules. Plant Physiol. 113, 127–135. 10.1104/pp.113.1.127 12223595 PMC158123

[B8] Auerbach G. Herrmann A. Bracher A. Bader G. Gütlich M. Fischer M.. (2000). Zinc plays a key role in human and bacterial GTP cyclohydrolase I. Proc. Natl. Acad. Sci. U.S.A. 97, 13567–13572. 10.1073/pnas.240463497 11087827 PMC17616

[B9] Bagley P. J. Selhub J. (2000). Analysis of folate form distribution by affinity followed by reversed- phase chromatography with electrical detection. Clin. Chem. 46, 404–411. 10702529

[B10] Balaghi M. Wagner C. (1993). DNA methylation in folate deficiency: use of CpG methylase. Biochem. Biophys. Res. Commun. 193, 1184–1190. 10.1006/bbrc.1993.1750 8323540

[B11] Barrueco J. R. Sirotnak F. (1991). Evidence for the facilitated transport of methotrexate polyglutamates into lysosomes derived from S180 cells. Basic properties and specificity for polyglutamate chain length. J. Biol. Chem. 266, 11732–11737. 1711038

[B12] Basset G. J. Quinlivan E. P. Ravanel S. Rébeillé F. Nichols B. P. Shinozaki K.. (2004a). Folate synthesis in plants: the p-aminobenzoate branch is initiated by a bifunctional PabA-PabB protein that is targeted to plastids. Proc. Natl. Acad. Sci. U.S.A. 101, 1496–1501. 10.1073/pnas.0308331100 14745019 PMC341757

[B13] Basset G. J. Ravanel S. Quinlivan E. P. White R. Giovannoni J. J. Rébeillé F.. (2004b). Folate synthesis in plants: the last step of the p-aminobenzoate branch is catalyzed by a plastidial aminodeoxychorismate lyase. Plant J. 40, 453–461. 10.1111/j.1365-313X.2004.02231.x 15500462

[B14] Basset G. Quinlivan E. P. Ziemak M. J. de la Garza R. Fischer M. Schiffmann S.. (2002). Folate synthesis in plants: the first step of the pterin branch is mediated by a unique bimodular GTP cyclohydrolase I. Proc. Natl. Acad. Sci. U.S.A. 99, 12489–12494. 10.1073/pnas.192278499 12221287 PMC129472

[B15] Baxter C. J. Redestig H. Schauer N. Repsilber D. Patil K. R. Nielsen J.. (2007). The metabolic response of heterotrophic Arabidopsis cells to oxidative stress. Plant Physiology 143, 312–325. 10.1104/pp.106.090431 17122072 PMC1761969

[B16] Bedhomme M. Hoffmann M. McCarthy E. A. Gambonnet B. Moran R. G. Rébeillé F.. (2005). Folate Metabolism in Plants: an Arabidopsis homolog of the mammalian mitochondrial folate transporter mediates folate import into chloroplasts. J. Biol. Chem. 280, 34823–34831. 10.1074/jbc.M506045200 16055441

[B17] Bekaert S. Storozhenko S. Mehrshahi P. Bennett M. J. Lambert W. Gregory J. F.. (2008). Folate biofortification in food plants. Trends Plant Sci. 13, 28–35. 10.1016/j.tplants.2007.11.001 18083061

[B18] Bello A. R. Nare B. Freedman D. Hardy L. Beverley S. M. (1994). PTR1: a reductase mediating salvage of oxidized pteridines and methotrexate resistance in the protozoan parasite Leishmania major. Proc. Natl. Acad. Sci. U.S.A. 91, 11442–11446. 10.1073/pnas.91.24.11442 7972081 PMC45247

[B19] Besson V. Rebeille F. Neuburger M. Douce R. Cossins E. (1993). Effects of tetrahydrofolate polyglutamates on the kinetic parameters of serine hydroxymethyltransferase and glycine decarboxylase from pea leaf mitochondria. Biochem. J. 292, 425–430. 10.1042/bj2920425 8503876 PMC1134226

[B20] Black M. T. Meyer D. Widger W. R. Cramer W. (1987). Light-regulated methylation of chloroplast proteins. J. Biol. Chem. 262, 9803–9807. 3597439

[B21] Blancquaert D. De Steur H. Gellynck X. Van Der Straeten D. (2014). Present and future of folate biofortification of crop plants. J. Exp. Bot. 65, 895–906. 10.1093/jxb/ert483 24574483

[B22] Blancquaert D. Steur H. Gellynck X. Van Der Straeten D.. (2017). Metabolic engineering of micronutrients in crop plants. Ann. NY Acad. Sci. 1390, 59–73. 10.1111/nyas.13274 27801945

[B23] Blancquaert D. Storozhenko S. Loizeau K. De Steur H. De Brouwer V. Viaene J. (2010). Folates and folic acid: from fundamental research toward sustainable health. Crit. Rev. Plant Sci. 29, 14–35. 10.1080/07352680903436283

[B24] Blancquaert D. Storozhenko S. Van Daele J. Stove C. Visser R. G. Lambert W.. (2013a). Enhancing pterin and para-aminobenzoate content is not sufficient to successfully biofortify potato tubers and *Arabidopsis thaliana* plants with folate. J. Exp. Bot. 64, 3899–3909. 10.1093/jxb/ert224 23956417

[B25] Blancquaert D. Van Daele J. Strobbe S. Kiekens F. Storozhenko S. De Steur H.. (2015). Improving folate (vitamin B9) stability in biofortified rice through metabolic engineering. Nat. Biotechnol. 33, 1076–1078. 10.1038/nbt.3358 26389575

[B26] Blancquaert D. Van Daele J. Storozhenko S. Stove C. Lambert W. Van Der Straeten D. (2013b). Rice folate enhancement through metabolic engineering has an impact on rice seed metabolism, but does not affect the expression of the endogenous folate biosynthesis genes. Plant Mol. Biol. 83, 329–349. 10.1007/s11103-013-0091-7 23771598

[B27] Blokhina O. Fagerstedt K. V. (2010). Reactive oxygen species and nitric oxide in plant mitochondria: origin and redundant regulatory systems. Physiol. Plant. 138, 447–462. 10.1111/j.1399-3054.2009.01340.x 20059731

[B28] Blundell G. Jones B. G. Rose F. A. Tudball N. (1996). Homocysteine mediated endothelial cell toxicity and its amelioration. Atherosclerosis 122, 163–172. 10.1016/0021-9150(95)05730-7 8769680

[B29] Bognar A. L. Osborne C. Shane B. Singer S. C. Ferone R. (1985). Folylpoly-gamma-glutamate synthetase-dihydrofolate synthetase. Cloning and high expression of the *Escherichia coli* folC gene and purification and properties of the gene product. J. Biol. Chem. 260, 5625–5630. 2985605

[B30] Bostom A. G. Shemin D. Lapane K. L. Nadeau M. R. Sutherland P. Chan J.. (1996). Folate status is the major determinant of fasting total plasma homocysteine levels in maintenance dialysis patients. Atherosclerosis 123, 193–202. 10.1016/0021-9150(96)05809-1 8782850

[B31] Bozzo G. G. Basset G. J. Naponelli V. Noiriel A. Gregory J. F. III Hanson A. D. (2008). Characterization of the folate salvage enzyme p-aminobenzoylglutamate hydrolase in plants. Phytochemistry 69, 29–37. 10.1016/j.phytochem.2007.06.031 17698154

[B32] Camara D. Bisanz C. Barette C. Van Daele J. Human E. Barnard B.. (2012). Inhibition of p-aminobenzoate and folate syntheses in plants and apicomplexan parasites by natural product rubreserine. J. Biol. Chem. 287, 22367–22376. 10.1074/jbc.M112.365833 22577137 PMC3381196

[B33] Camara D. Richefeu-Contesto C. Gambonnet B. Dumas R. Rébeillé F. (2011). The synthesis of pABA: coupling between the glutamine amidotransferase and aminodeoxychorismate synthase domains of the bifunctional aminodeoxychorismate synthase from *Arabidopsis thaliana*. Arch. Biochem. Biophys. 505, 83–90. 10.1016/j.abb.2010.09.010 20851095

[B34] Cano M. J. Ayala A. Murillo M. L. Carreras O. (2001). Protective effect of folic acid against oxidative stress produced in 21-day postpartum rats by maternal-ethanol chronic consumption during pregnancy and lactation period. Free Radic. Res. 34, 1–8. 10.1080/10715760100300011 11234991

[B35] Cella R. Crosti P. Nielsen E. Parisi B. (1983). Biochemical basis of different sensitivity to methotrexate in *Daucus carota* and *Oryza sativa* cell cultures. J. Exp. Bot. 34, 1189–1195. 10.1093/jxb/34.9.1189

[B36] Chabouté M. E. Combettes B. Clément B. Gigot C. Philipps G. (1998). Molecular characterization of tobacco ribonucleotide reductase RNR1 and RNR2 cDNAs and cell cycle-regulated expression in synchronized plant cells. Plant Mol. Biol. 38, 797–806. 10.1023/A:1006083318906 9862497

[B37] Chen L. Chan S. Y. Cossins E. A. (1997). Distribution of folate derivatives and enzymes for synthesis of 10-formyltetrahydrofolate in cytosolic and mitochondrial fractions of pea leaves. Plant Physiol. 115, 299–309. 10.1104/pp.115.1.299 12223808 PMC158486

[B38] Clandinin M. T. Cossins E. A. (1974). Methionine biosynthesis in isolated *Pisum sativum* mitochondria. Phytochemistry 13, 585–591. 10.1016/S0031-9422(00)91356-6

[B39] Cossins E. A. (1987). Folate biochemistry and the metabolism of one-carbon units. Biochem. Plants 11, 317–353. 10.1016/b978-0-12-675411-7.50015-x

[B40] Cossins E. A. (2000). The fascinating world of folate and one-carbon metabolism. Botany 78:691 10.1139/cjb-78-6-691

[B41] Cox K. Robertson D. Fites R. (1999). Mapping and expression of a bifunctional thymidylate synthase, dihydrofolate reductase gene from maize. Plant Mol. Biol. 41, 733–739. 10.1023/A:1006324328355 10737138

[B42] Crider K. S. Yang T. P. Berry R. J. Bailey L. B. (2012). Folate and DNA methylation: a review of molecular mechanisms and the evidence for folate's role. Adv. Nutrit 3, 21–38. 10.3945/an.111.000992 22332098 PMC3262611

[B43] Crosti P. (1981). Effect of folate analogues on the activity of dihydrofolate reductases and on the growth of plant organisms. J. Exp. Bot. 32, 717–723. 10.1093/jxb/32.4.717

[B44] Dallas W. S. Gowen J. E. Ray P. H. Cox M. J. Dev I. K. (1992). Cloning, sequencing, and enhanced expression of the dihydropteroate synthase gene of *Escherichia coli* MC4100. J. Bacteriol. 174, 5961–5970. 10.1128/jb.174.18.5961-5970.1992 1522070 PMC207134

[B45] De Brouwer V. Storozhenko S. Stove C. P. Van Daele J. Van Der Straeten D. Lambert W. E. (2010). Ultra-performance liquid chromatography-tandem mass spectrometry (UPLC-MS/MS) for the sensitive determination of folates in rice. J. Chromatogr. B 878, 509–513. 10.1016/j.jchromb.2009.12.032 20061193

[B46] De Brouwer V. Storozhenko S. Van De Steene J. C. Wille S. M. Stove C. P. Van Der Straeten D.. (2008). Optimisation and validation of a liquid chromatography-tandem mass spectrometry method for folates in rice. J. Chromatogr. A 1215, 125–132. 10.1016/j.chroma.2008.11.004 19026416

[B47] De Brouwer V. Zhang G.-F. Storozhenko S. Van Der Straeten D. Lambert W. E. (2007). pH stability of individual folates during critical sample preparation steps in prevision of the analysis of plant folates. Phytochem. Anal. 18, 496–508. 10.1002/pca.1006 17624887

[B48] de La Garza R. I. D. Gregory J. F. Hanson A. D. (2007). Folate biofortification of tomato fruit. Proc. Natl. Acad. Sci. U.S.A. 104, 4218–4222. 10.1073/pnas.0700409104 17360503 PMC1810332

[B49] del Río L. A. Sandalio L. M. Corpas F. J. Palma J. M. Barroso J. B. (2006). Reactive oxygen species and reactive nitrogen species in peroxisomes. Production, scavenging, and role in cell signaling. Plant Physiol. 141, 330–335. 10.1104/pp.106.078204 16760483 PMC1475433

[B50] DeSouza L. Shen Y. Bognar A. L. (2000). Disruption of cytoplasmic and mitochondrial folylpolyglutamate synthetase activity in *Saccharomyces cerevisiae*. Arch. Biochem. Biophys. 376, 299–312. 10.1006/abbi.2000.1741 10775416

[B51] Dhitavat S. Ortiz D. Rogers E. Rivera E. Shea T. B. (2005). Folate, vitamin E, and acetyl-L-carnitine provide synergistic protection against oxidative stress resulting from exposure of human neuroblastoma cells to amyloid-beta. Brain Res. 1061, 114–117. 10.1016/j.brainres.2005.05.074 16256963

[B52] Diaz C. Lemaître T. Christ A. Azzopardi M. Kato Y. Sato F.. (2008). Nitrogen recycling and remobilization are differentially controlled by leaf senescence and development stage in Arabidopsis under low nitrogen nutrition. Plant Physiol. 147, 1437–1449. 10.1104/pp.108.119040 18467460 PMC2442554

[B53] Diaz C. Saliba-Colombani V. Loudet O. Belluomo P. Moreau L. Daniel-Vedele F.. (2006). Leaf yellowing and anthocyanin accumulation are two genetically independent strategies in response to nitrogen limitation in *Arabidopsis thaliana*. Plant Cell Physiol. 47, 74–83. 10.1093/pcp/pci225 16284408

[B54] Díaz de la Garza R. Quinlivan E. P. Klaus S. M. Basset G. J. Gregory J. F. III Hanson A. D. (2004). Folate biofortification in tomatoes by engineering the pteridine branch of folate synthesis. Proc. Natl. Acad. Sci. U.S.A. 101, 13720–13725. 10.1073/pnas.0404208101 15365185 PMC518823

[B55] Dixon K. H. Lanpher B. C. Chiu J. Kelley K. Cowan K. H. (1994). A novel cDNA restores reduced folate carrier activity and methotrexate sensitivity to transport deficient cells. J. Biol. Chem. 269, 17–20. 8276792

[B56] Doherty R. F. Beecher G. R. (2003). A method for the analysis of natural and synthetic folate in foods. J. Agric. Food Chem. 51, 354–361. 10.1021/jf0259056 12517095

[B57] Douce R. Bourguignon J. Neuburger M. Rébeillé F. (2001). The glycine decarboxylase system: a fascinating complex. Trends Plant Sci. 6, 167–176. 10.1016/S1360-1385(01)01892-1 11286922

[B58] Edman J. C. Goldstein A. L. Erbe J. G. (1993). Para-aminobenzoate synthase gene of *Saccharomyces cerevisiae* encodes a bifunctional enzyme. Yeast 9, 669–675. 10.1002/yea.320090613 8346682

[B59] Eichel J. González J. C. Hotze M. Matthews R. G. Schröder J. (1995). Vitamin-B12-Independent Methionine Synthase from a Higher Plant (Catharanthus Roseus). Eur. J. Biochem. 230, 1053–1058. 10.1111/j.1432-1033.1995.tb20655.x 7601135

[B60] Engstrom E. M. Andersen C. M. Gumulak-Smith J. Hu J. Orlova E. Sozzani R.. (2011). Arabidopsis homologs of the petunia hairy meristem gene are required for maintenance of shoot and root indeterminacy. Plant Physiol. 155, 735–750. 10.1104/pp.110.168757 21173022 PMC3032463

[B61] Eudes A. Bozzo G. G. Waller J. C. Naponelli V. Lim E. K. Bowles D. J.. (2008a). Metabolism of the Folate Precursor p-Aminobenzoate in Plants Glucose Ester Formation And Vacuolar Storage. J. Biol. Chem. 283, 15451–15459. 10.1074/jbc.M709591200 18385129 PMC2397476

[B62] Eudes A. Erkens G. B. Slotboom D. J. Rodionov D. A. Naponelli V. Hanson A. D. (2008b). Identification of genes encoding the folate-and thiamine-binding membrane proteins in Firmicutes. J. Bacteriol. 190, 7591–7594. 10.1128/JB.01070-08 18776013 PMC2576666

[B63] Eudes A. Kunji E. R. Noiriel A. Klaus S. M. Vickers T. J. Beverley S. M.. (2010). Identification of transport-critical residues in a folate transporter from the folate-biopterin transporter (FBT) family. J. Biol. Chem. 285, 2867–2875. 10.1074/jbc.M109.063651 19923217 PMC2807340

[B64] Fan J. Ye J. Kamphorst J. J. Shlomi T. Thompson C. B. Rabinowitz J. D. (2014). Quantitative flux analysis reveals folate-dependent NADPH production. Nature. 510:298. 10.1038/nature13236 24805240 PMC4104482

[B65] Fazili Z. Pfeiffer C. M. Zhang M. Jain R. (2005). Erythrocyte folate extraction and quantitative determination by liquid chromatography-tandem mass spectrometry: comparison of results with microbiologic assay. Clin. Chem. 51, 2318–2325. 10.1373/clinchem.2005.053801 16214826

[B66] Fenech M. (2001). The role of folic acid and vitamin B12 in genomic stability of human cells. Mut. Res. Fundamen. Mol. Mech. Mutagen. 475, 57–67. 10.1016/S0027-5107(01)00079-3 11295154

[B67] Fitzpatrick T. B. Basset G. J. Borel P. Carrari F. DellaPenna D. Fraser P. D.. (2012). Vitamin deficiencies in humans: can plant science help? Plant Cell. 24, 395–414. 10.1105/tpc.111.093120 22374394 PMC3315223

[B68] Foyer C. H. Harbinson J. C. (1994). Oxygen metabolism and the regulation of photosynthetic electron transport, in Causes of Photooxidative Stress and Amelioration of Defense Systems in Plant, eds Foyer C. H. Mullineaux P. M. (Boca Raton, FL: CRC Press), 1–42.

[B69] Freemantle S. J. Taylor S. M. Krystal G. Moran R. G. (1995). Upstream organization of and multiple transcripts from the human folylpoly–glutamate synthetase gene. J. Biol. Chem. 270, 9579–9584. 10.1074/jbc.270.16.9579 7721888

[B70] Freisleben A. Schieberle P. Rychlik M. (2003a). Comparison of folate quantification in foods by high-performance liquid chromatography-fluorescence detection to that by stable isotope dilution assays using high-performance liquid chromatography-tandem mass spectrometry. Anal. Biochem. 315, 247–255. 10.1016/S0003-2697(03)00029-0 12689834

[B71] Freisleben A. Schieberle P. Rychlik M. (2003b). Specific and sensitive quantification of folate vitamers in foods by stable isotope dilution assays using high-performance liquid chromatography-tandem mass spectrometry. Anal. Bioanal. Chem. 376, 149–156. 10.1007/s00216-003-1844-y 12698226

[B72] Gambonnet B. Jabrin S. Ravanel S. Karan M. Douce R. Rebeille F. (2001). Folate distribution during higher plant development. J. Sci. Food Agric. 81, 835–841. 10.1002/jsfa.870

[B73] Garratt L. C. Ortori C. A. Tucker G. A. Sablitzky F. Bennett M. J. Barrett D. A. (2005). Comprehensive metabolic profiling of mono- and polyglutamated folates and their precursors in plant and animal tissue using liquid chromatography/negative ion electrospray ionisation tandem mass spectrometry. Rapid Commun. Mass Spectrom. 19, 2390–2398. 10.1002/rcm.2074 16047318

[B74] Gillies S. A. McIntosh S. R. Henry R. J. (2008). A cereal crop with enhanced folate: rice transgenic for wheat HPPK/DHPS, in ComBio 2008 (Canberra, ACT; Lismore, NSW: Southern Cross Plant Science, Centre for Plant Conservation Genetics).

[B75] Goldman I. D. Lichtenstein N. S. Oliverio V. T. (1968). Carrier-mediated transport of the folic acid analogue, methotrexate, in the L1210 leukemia cell. J. Biol. Chem. 243, 5007–5017. 5303004

[B76] Goyer A. Collakova E. de la Garza R. D. Quinlivan E. P. Williamson J. Gregory J. F. III. (2005). 5-Formyltetrahydrofolate is an inhibitory but well tolerated metabolite in Arabidopsis leaves. J. Biol. Chem. 280, 26137–26142. 10.1074/jbc.M503106200 15888445

[B77] Goyer A. Illarionova V. Roje S. Fischer M. Bacher A. Hanson A. D. (2004). Folate biosynthesis in higher plants. cDNA cloning, heterologous expression, and characterization of dihydroneopterin aldolases. Plant Physiol. 135, 103–111. 10.1104/pp.103.038430 15107504 PMC429337

[B78] Goyer A. Navarre D. A. (2007). Determination of folate concentrations in diverse potato germplasm using a trienzyme extraction and a microbiological assay. J. Agric. Food Chem. 55, 3523–3528. 10.1021/jf063647x 17419642

[B79] Goyer A. Sweek K. (2011). Genetic diversity of thiamin and folate in primitive cultivated and wild potato (Solanum) species. J. Agric. Food Chem. 59, 13072–13080. 10.1021/jf203736e 22088125

[B80] Green J. M. Merkel W. K. Nichols B. P. (1992). Characterization and sequence of *Escherichia coli* pabC, the gene encoding aminodeoxychorismate lyase, a pyridoxal phosphate-containing enzyme. J. Bacteriol. 174, 5317–5323. 10.1128/jb.174.16.5317-5323.1992 1644759 PMC206368

[B81] Gregory J. F. (1989). Chemical and nutritional aspects of folate research: analytical procedures, methods of folate synthesis, stability, and bioavailability of dietary folates. Adv. Food Nutr. Res. 33, 1–101. 10.1016/S1043-4526(08)60126-6 2697230

[B82] Güldener U. Koehler G. J. Haussmann C. Bacher A. Kricke J. Becher D.. (2004). Characterization of the *Saccharomyces cerevisiae* Fol1 protein: starvation for C1 carrier induces pseudohyphal growth. Mol. Biol. Cell. 15, 3811–3828. 10.1091/mbc.E03-09-0680 15169867 PMC491839

[B83] Gutzeit D. Mönch S. Jerz G. Winterhalter P. Rychlik M. (2008). Folate content in sea buckthorn berries and related products (*Hippophae rhamnoides* L. ssp. rhamnoides): LC-MS/MS determination of folate vitamer stability influenced by processing and storage assessed by stable isotope dilution assay. Anal. Bioanal. Chem. 391, 211–219. 10.1007/s00216-008-1905-3 18278485

[B84] Hanson A. D. Roje S. (2001). One-carbon metabolism in higher plants. Annu. Rev. Plant Biol. 52, 119–137. 10.1146/annurev.arplant.52.1.119 11337394

[B85] Heyno E. Mary V. Schopfer P. Krieger-Liszkay A. (2011). Oxygen activation at the plasma membrane: relation between superoxide and hydroxyl radical production by isolated membranes. Planta 234, 35–45. 10.1007/s00425-011-1379-y 21359959

[B86] Ho P. I. Ashline D. Dhitavat S. Ortiz D. Collins S. C. Shea T. B.. (2003). Folate deprivation induces neurodegeneration: roles of oxidative stress and increased homocysteine. Neurobiol. Dis. 14, 32–42. 10.1016/S0969-9961(03)00070-6 13678664

[B87] Hoang N. Bouly J. P. Ahmad M. (2008). Evidence of a light-sensing role for folate in Arabidopsis cryptochrome blue-light receptors. Mol. Plant. 1, 68–74. 10.1093/mp/ssm008 20031915

[B88] Hossain T. Rosenberg I. Selhub J. Kishore G. Beachy R. Schubert K. (2004). Enhancement of folates in plants through metabolic engineering. Proc. Natl. Acad. Sci. U.S.A. 101, 5158–5163. 10.1073/pnas.0401342101 15044686 PMC387390

[B89] Huang R. F. Hsu Y. C. Lin H. L. Yang F. L. (2001). Folate depletion and elevated plasma homocysteine promote oxidative stress in rat livers. J. Nutr. 131, 33–38. 11208935 10.1093/jn/131.1.33

[B90] Hussein M. J. Green J. M. Nichols B. P. (1998). Characterization of Mutations That Allow p-Aminobenzoyl-Glutamate Utilization by *Escherichia coli*. J. Bacteriol. 180, 6260–6268. 9829935 10.1128/jb.180.23.6260-6268.1998 PMC107711

[B91] Hutchinson M. L. Jones M. Nixon P. F. (2000). Stability of labile folate is enhanced by binding milk folate-binding protein. Dairy Ingred. Sci. 2000 Conf. (Abstract), 55, 98.

[B92] Hyun T. H. Tamura T. (2005). Trienzyme extraction in combination with microbiologic assay in food folate analysis: an updated review. Exp. Biol. Med. 230, 444–454. 10.1177/153537020523000702 15985619

[B93] Isegawa Y. Watanabe F. Kitaoka S. Nakano Y. (1993). Subcellular distribution of cobalamin-dependent methionine synthase in *Euglena gracilis* Z. Phytochemistry 35, 59–61. 10.1016/S0031-9422(00)90509-0

[B94] Ishikawa T. Machida C. Yoshioka Y. Kitano H. Machida Y. (2003). The GLOBULAR ARREST1 gene, which is involved in the biosynthesis of folates, is essential for embryogenesis in *Arabidopsis thaliana*. Plant J. 33, 235–244. 10.1046/j.1365-313X.2003.01621.x 12535338

[B95] Jabrin S. Ravanel S. Gambonnet B. Douce R. Rébeillé F. (2003). One-carbon metabolism in plants. Regulation of tetrahydrofolate synthesis during germination and seedling development. Plant Physiol. 131, 1431–1439. 10.1104/pp.016915 12644692 PMC166902

[B96] Jacob R. A. Wu M. M. Henning S. M. Swendseid M. E. (1994). Homocysteine increases as folate decreases in plasma of healthy men during short-term dietary folate and methyl group restriction. J. Nutr. 124, 1072–1080. 8027858 10.1093/jn/124.7.1072

[B97] Jägerstad M. Jastrebova J. (2013). Occurrence, stability, and determination of formyl folates in foods. J. Agric. Food Chem. 61, 9758–9768. 10.1021/jf4028427 24033320

[B98] James T. Y. Boulianne R. P. Bottoli A. P. Granado J. D. Aebi M. Kües U. (2002). The pab1 gene of Coprinus cinereus encodes a bifunctional protein for para-aminobenzoic acid (PABA) synthesis: implications for the evolution of fused PABA synthases. J. Basic Microbiol. 42, 91–103. 10.1002/1521-4028(200205)42:2<91::AID-JOBM91>3.0.CO;2-8 11981873

[B99] Jencks D. A. Mathews R. G. (1987). Allosteric inhibition of methylenetetrahydrofolate reductase by adenosylmethionine. Effects of adenosylmethionine and NADPH on the equilibrium between active and inactive forms of the enzyme and on the kinetics of approach to equilibrium. J. Biol. Chem. 262, 2485–2493. 3818603

[B100] Jhaveri M. S. Wagner C. Trepel J. B. (2001). Impact of extracellular folate levels on global gene expression. Mol. Pharmacol. 60, 1288–1295. 10.1124/mol.60.6.1288 11723236

[B101] Jiang L. Liu Y. Sun H. Han Y. Li J. Li C.. (2013). The mitochondrial folylpolyglutamate synthetase gene is required for nitrogen utilization during early seedling development in Arabidopsis. Plant Physiol. 161, 971–989. 10.1104/pp.112.203430 23129207 PMC3561033

[B102] Jones M. L. Nixon P. F. (2002). Tetrahydrofolates are greatly stabilized by binding to bovine milk folate-binding protein. J. Nutr. 132, 2690–2694. 12221230 10.1093/jn/132.9.2690

[B103] Kamen B. Wang M. T. Streckfuss A. J. Peryea X. Anderson R. G. (1988). Delivery of folates to the cytoplasm of MA104 cells is mediated by a surface membrane receptor that recycles. J. Biol. Chem. 263, 13602–13609. 3417674

[B104] Kim H. N. Kim Y. K. Lee I. K. Yang D. H. Lee J. J. Shin M. H.. (2009). Association between polymorphisms of folate-metabolizing enzymes and hematological malignancies. Leuk. Res. 33, 82–87. 10.1016/j.leukres.2008.07.026 18774170

[B105] Kim Y.-I. (2003). Role of folate in colon cancer development and progression. J. Nutrit. 133, 3731S–3739S. 14608107 10.1093/jn/133.11.3731S

[B106] Kirk C. D. Chen L. Imeson H. C. Cossins E. A. (1995). A 5, 10-methylenetetrahydrofolate dehydrogenase: 5, 10-methenyltetrahydrofolate cyclohydrolase protein from *Pisum sativum*. Phytochemistry 39, 1309–1317. 10.1016/0031-9422(95)97864-6

[B107] Kirk C. D. Imeson H. C. Zheng L.-L. Cossins E. A. (1994). The affinity of pea cotyledon 10-formyltetrahydrofolate synthetase for polyglutamate substrates. Phytochemistry 35, 291–296. 10.1016/S0031-9422(00)94750-2

[B108] Klaus S. M. Kunji E. R. Bozzo G. G. Noiriel A. de La Garza R. D. Basset G. J.. (2005a). Higher plant plastids and cyanobacteria have folate carriers related to those of trypanosomatids. J. Biol. Chem. 280, 38457–38463. 10.1074/jbc.M507432200 16162503

[B109] Klaus S. M. Wegkamp A. Sybesma W. Hugenholtz J. Gregory J. F. III Hanson A. D. (2005b). A nudix enzyme removes pyrophosphate from dihydroneopterin triphosphate in the folate synthesis pathway of bacteria and plants. J. Biol. Chem. 280, 5274–5280. 10.1074/jbc.M413759200 15611104

[B110] Konings E. J. Roomans H. H. Dorant E. Goldbohm R. A. Saris W. H. van den Brandt P. A. (2001). Folate intake of the Dutch population according to newly established liquid chromatography data for foods. Am. J. Clin. Nutr. 73, 765–776. 11273852 10.1093/ajcn/73.4.765

[B111] Kramer D. M. Evans J. R. (2011). The importance of energy balance in improving photosynthetic productivity. Plant Physiol. 155, 70–78. 10.1104/pp.110.166652 21078862 PMC3075755

[B112] Kruger N. J. von Schaewen A. (2003). The oxidative pentose phosphate pathway: structure and organisation. Curr. Opin. Plant Biol. 6, 236–246. 10.1016/S1369-5266(03)00039-6 12753973

[B113] Lee D. Y. Northrop J. P. Kuo M. H. Stallcup M. R. (2006). Histone H3 lysine 9 methyltransferase G9a is a transcriptional coactivator for nuclear receptors. J. Biol. Chem. 281, 8476–8485. 10.1074/jbc.M511093200 16461774 PMC1770944

[B114] Lemaître T. Gaufichon L. Boutet-Mercey S. Christ A. Masclaux-Daubresse C. (2008). Enzymatic and metabolic diagnostic of nitrogen deficiency in *Arabidopsis thaliana* Wassileskija accession. Plant Cell Physiol. 49, 1056–1065. 10.1093/pcp/pcn081 18508804

[B115] Lemley C. Yan S. Dole V. S. Madhubala R. Cunningham M. L. Beverley S. M.. (1999). The *Leishmania donovani* LD1 locus gene ORFG encodes a biopterin transporter (BT1). Mol. Biochem. Parasitol. 104, 93–105. 10.1016/S0166-6851(99)00132-2 10589984

[B116] Liu J. Lynne Ward R. (2010). 4 Folate and One-Carbon Metabolism and Its Impact on Aberrant DNA Methylation in Cancer. Adv. Genet. 71:79. 10.1016/b978-0-12-380864-6.00004-3 20933127

[B117] Loizeau K. De Brouwer V. Gambonnet B. Yu A. Renou J. P. Van Der Straeten D.. (2008). A genome-wide and metabolic analysis determined the adaptive response of Arabidopsis cells to folate depletion induced by methotrexate. Plant Physiol. 148, 2083–2095. 10.1104/pp.108.130336 18931140 PMC2593662

[B118] Loizeau K. Gambonnet B. Zhang G. F. Curien G. Jabrin S. Van Der Straeten D.. (2007). Regulation of one-carbon metabolism in Arabidopsis: the N-terminal regulatory domain of cystathionine γ-synthase is cleaved in response to folate starvation. Plant Physiol. 145, 491–503. 10.1104/pp.107.105379 17720756 PMC2048731

[B119] Luo M. Orsi R. Patrucco E. Pancaldi S. Cella R. (1997). Multiple transcription start sites of the carrot dihydrofolate reductase-thymidylate synthase gene, and sub-cellular localization of the bifunctional protein. Plant Mol. Biol. 33, 709–722. 10.1023/A:1005798207693 9132062

[B120] Luo M. Piffanelli P. Rastelli L. Cella R. (1993). Molecular cloning and analysis of a cDNA coding for the bifunctional dihydrofolate reductase-thymidylate synthase of *Daucus carota*. Plant Mol. Biol. 22, 427–435. 10.1007/BF00015973 8329682

[B121] Martin J. I. Landen W. O. Jr. Soliman A. G. Eitenmiller R. R. (1990). Application of a tri-enzyme extraction for total folate determination in foods. J. AOAC Int. 73, 805–808. 2125600

[B122] Martin T. Oswald O. Graham I. A. (2002). Arabidopsis seedling growth, storage lipid mobilization, and photosynthetic gene expression are regulated by carbon: nitrogen availability. Plant Physiol. 128, 472–481. 10.1104/pp.010475 11842151 PMC148910

[B123] McCarthy E. A. Titus S. A. Taylor S. M. Jackson-Cook C. Moran R. G. (2004). A mutation inactivating the mitochondrial inner membrane folate transporter creates a glycine requirement for survival of Chinese hamster cells. J. Biol. Chem. 279, 33829–33836. 10.1074/jbc.M403677200 15140890

[B124] McIntosh S. R. Brushett D. Henry R. J. (2008). GTP cyclohydrolase 1 expression and folate accumulation in the developing wheat seed. J. Cereal Sci. 48, 503–512. 10.1016/j.jcs.2007.11.008

[B125] McIntosh S. R. Henry R. J. (2008). Genes of folate biosynthesis in wheat. J. Cereal Sci. 48, 632–638. 10.1016/j.jcs.2008.02.007

[B126] Mehrshahi P. Gonzalez-Jorge S. Akhtar T. A. Ward J. L. Santoyo-Castelazo A. Marcus S. E.. (2010). Functional analysis of folate polyglutamylation and its essential role in plant metabolism and development. Plant J. 64, 267–279. 10.1111/j.1365-313X.2010.04336.x 21070407

[B127] Meng H. Jiang L. Xu B. Guo W. Li J. Zhu X.. (2014). Arabidopsis plastidial folylpolyglutamate synthetase is required for seed reserve accumulation and seedling establishment in darkness. PLoS ONE 9:e101905. 10.1371/journal.pone.0101905 25000295 PMC4084893

[B128] Moffatt B. A. Weretilnyk E. A. (2001). Sustaining S-adenosyl-l-methionine-dependent methyltransferase activity in plant cells. Physiol. Plant. 113, 435–442. 10.1034/j.1399-3054.2001.1130401.x

[B129] Møller I. M. Rasmusson A. G. (1998). The role of NADP in the mitochondrial matrix. Trends Plant Sci. 3, 21–27. 10.1016/S1360-1385(97)01156-4

[B130] Molloy A. M. (2012). Genetic aspects of folate metabolism, in Water Soluble, Vitamins, ed Stanger O. (Berlin: Springer), 105–130. 10.1007/978-94-007-2199-9_7

[B131] Mouillon J. M. Aubert S. Bourguignon J. Gout E. Douce R. Rébeillé F. (1999). Glycine and serine catabolism in non-photosynthetic higher plant cells: their role in C1 metabolism. Plant J. 20, 197–205. 10.1046/j.1365-313x.1999.00591.x 10571879

[B132] Mouillon J. M. Ravanel S. Douce R. Rébeillé F. (2002). Folate synthesis in higher-plant mitochondria: coupling between the dihydropterin pyrophosphokinase and the dihydropteroate synthase activities. Biochem. J. 363, 313–319. 10.1042/bj3630313 11931659 PMC1222480

[B133] Naqvi S. Zhu C. Farre G. Ramessar K. Bassie L. Breitenbach J.. (2009). Transgenic multivitamin corn through biofortification of endosperm with three vitamins representing three distinct metabolic pathways. Proc. Natl. Acad. Sci. U.S.A. 106, 7762–7767. 10.1073/pnas.0901412106 19416835 PMC2683132

[B134] Nar H. Huber R. Auerbach G. Fischer M. Hösl C. Ritz H.. (1995). Active site topology and reaction mechanism of GTP cyclohydrolase I. Proc. Natl. Acad. Sci. U.S.A. 92, 12120–12125. 10.1073/pnas.92.26.12120 8618856 PMC40308

[B135] Nardese V. Gütlich M. Brambilla A. Carbone M. L. (1996). Disruption of the GTP-Cyclohydrolase I Gene In*Saccharomyces cerevisiae*. Biochem. Biophys. Res. Commun. 218, 273–279. 10.1006/bbrc.1996.0048 8573145

[B136] Navarrete O. Van Daele J. Stove C. Lambert W. Storozhenko S. Van Der Straeten D. (2013). Isolation and characterisation of an antifolate insensitive (afi1) mutant of *Arabidopsis thaliana*. Plant Biol. 15, 37–44. 10.1111/j.1438-8677.2012.00602.x 22672761

[B137] Ndaw S. Bergaentzlé M. Aoudé-Werner D. Lahély S. Hasselmann C. (2001). Determination of folates in foods by high-performance liquid chromatography with fluorescence detection after precolumn conversion to 5-methyltetrahydrofolates. J. Chromatogr. A 928, 77–90. 10.1016/S0021-9673(01)01129-3 11589473

[B138] Neilson K. A. Mariani M. Haynes P. A. (2011). Quantitative proteomic analysis of cold-responsive proteins in rice. Proteomics 11, 1696–1706. 10.1002/pmic.201000727 21433000

[B139] Nelson B. C. Pfeiffer C. M. Margolis S. A. Nelson C. P. (2004). Solid-phase extraction-electrospray ionization mass spectrometry for the quantification of folate in human plasma or serum. Anal. Biochem. 325, 41–51. 10.1016/j.ab.2003.10.009 14715283

[B140] Neuburger M. Rébeillé F. Jourdain A. Nakamura S. Douce R. (1996). Mitochondria are a major site for folate and thymidylate synthesis in plants. J. Biol. Chem. 271, 9466–9472. 10.1074/jbc.271.16.9466 8621617

[B141] Nichols B. P. Seibold A. M. Doktor S. Z. (1989). para-aminobenzoate synthesis from chorismate occurs in two steps. J. Biol. Chem. 264, 8597–8601. 2656685

[B142] Noctor G. Foyer C. H. (1998). Ascorbate and glutathione: keeping active oxygen under control. Annu. Rev. Plant Biol. 49, 249–279. 10.1146/annurev.arplant.49.1.249 15012235

[B143] Noiriel A. Naponelli V. Bozzo G. G. Gregory J. F. III Hanson A. D. (2007a). Folate salvage in plants: pterin aldehyde reduction is mediated by multiple non-specific aldehyde reductases. Plant J. 51, 378–389. 10.1111/j.1365-313X.2007.03143.x 17550420

[B144] Noiriel A. Naponelli V. Gregory J. F. III Hanson A. D. (2007b). Pterin and folate salvage. Plants and *Escherichia coli* lack capacity to reduce oxidized pterins. Plant Physiol. 143, 1101–1109. 10.1104/pp.106.093633 17220358 PMC1820931

[B145] Nunes A. C. Kalkmann D. C. Aragao F. J. (2009). Folate biofortification of lettuce by expression of a codon optimized chicken GTP cyclohydrolase I gene. Transgenic Res. 18, 661–667. 10.1007/s11248-009-9256-1 19322672

[B146] Orsomando G. Bozzo G. G. Garza R. D. Basset G. J. Quinlivan E. P. Naponelli V.. (2006). Evidence for folate-salvage reactions in plants. Plant J. 46, 426–435. 10.1111/j.1365-313X.2006.02685.x 16623903

[B147] Orsomando G. de la Garza R. D. Green B. J. Peng M. Rea P. A. Ryan T. J.. (2005). Plant γ-Glutamyl Hydrolases and Folate Polyglutamates characterization, compartmentation, and co-occurrence in vacuoles. J. Biol. Chem. 280, 28877–28884. 10.1074/jbc.M504306200 15961386

[B148] Patring J. D. Jastrebova J. A. (2007). Application of liquid chromatography-electrospray ionisation mass spectrometry for determination of dietary folates: effects of buffer nature and mobile phase composition on sensitivity and selectivity. J. Chromatogr. A. 1143, 72–82. 10.1016/j.chroma.2006.12.079 17210159

[B149] Pfeiffer C. M. Rogers L. M. Gregory J. F. (1997). Determination of folate in cereal-grain food products using trienzyme extraction and combined affinity and reversed-phase liquid chromatography. J. Agric. Food Chem. 45, 407–413. 10.1021/jf960633q

[B150] Pogribny I. P. James S. J. (2002). *De novo* methylation of the p16INK4A gene in early preneoplastic liver and tumors induced by folate/methyl deficiency in rats. Cancer Lett. 187, 69–75. 10.1016/S0304-3835(02)00408-1 12359353

[B151] Powell J. J. Fitzgerald T. L. Stiller J. Berkman P. J. Gardiner D. M. Manners J. M.. (2016). The defence-associated transcriptome of hexaploid wheat displays homoeolog expression and induction bias. Plant Biotechnol. J. 15, 533–543. 10.1111/pbi.12651 27735125 PMC5362679

[B152] Prabhu V. Chatson K. B. Abrams G. D. King J. (1996). 13C nuclear magnetic resonance detection of interactions of serine hydroxymethyltransferase with C1-tetrahydrofolate synthase and glycine decarboxylase complex activities in Arabidopsis. Plant Physiol. 112, 207–216. 10.1104/pp.112.1.207 8819325 PMC157939

[B153] Prabhu V. Chatson K. B. Lui H. Abrams G. D. King J. (1998). Effects of sulfanilamide and methotrexate on 13C fluxes through the glycine decarboxylase/serine hydroxymethyltransferase enzyme system in Arabidopsis. Plant Physiol. 116, 137–144. 10.1104/pp.116.1.137 9449840 PMC35151

[B154] Quinlivan E. P. Hanson A. D. Gregory J. F. (2006). The analysis of folate and its metabolic precursors in biological samples. Anal. Biochem. 348, 163–184. 10.1016/j.ab.2005.09.017 16266679

[B155] Quinlivan E. P. Roje S. Basset G. Shachar-Hill Y. Gregory J. F. Hanson A. D. (2003). The folate precursor p-aminobenzoate is reversibly converted to its glucose ester in the plant cytosol. J. Biol. Chem. 278, 20731–20737. 10.1074/jbc.M302894200 12668665

[B156] Rader J. I. Weaver C. M. Angyal G. (1998). Use of a microbiological assay with tri-enzyme extraction for measurement of pre-fortification levels of folates in enriched cereal-grain products. Food Chem. 62, 451–465. 10.1016/S0308-8146(98)00089-2

[B157] Raichaudhuri A. Peng M. Naponelli V. Chen S. Sánchez-Fernández R. Gu H.. (2009). Plant vacuolar ATP-binding cassette transporters that translocate folates and antifolates *in vitro* and contribute to antifolate tolerance *in vivo*. J. Biol. Chem. 284, 8449–8460. 10.1074/jbc.M808632200 19136566 PMC2659203

[B158] Rasmusson A. G. Møller I. M. (1990). NADP-utilizing enzymes in the matrix of plant mitochondria. Plant Physiol. 94, 1012–1018. 10.1104/pp.94.3.1012 16667790 PMC1077335

[B159] Ravanel S. Block M. A. Rippert P. Jabrin S. Curien G. Rébeillé F.. (2004). Methionine metabolism in plants chloroplasts are autonomous for de novo methionine synthesis and can import s-adenosylmethionine from the cytosol. J. Biol. Chem. 279, 22548–22557. 10.1074/jbc.M313250200 15024005

[B160] Ravanel S. Cherest H. Jabrin S. Grunwald D. Surdin-Kerjan Y. Douce R.. (2001). Tetrahydrofolate biosynthesis in plants: molecular and functional characterization of dihydrofolate synthetase and three isoforms of folylpolyglutamate synthetase in *Arabidopsis thaliana*. Proc. Natl. Acad. Sci. U.S.A. 98, 15360–15365. 10.1073/pnas.261585098 11752472 PMC65034

[B161] Rébeillé F. Macherel D. Mouillon J. M. Garin J. Douce R. (1997). Folate biosynthesis in higher plants: purification and molecular cloning of a bifunctional 6-hydroxymethyl-7, 8-dihydropterin pyrophosphokinase/7, 8-dihydropteroate synthase localized in mitochondria. EMBO J. 16, 947–957. 10.1093/emboj/16.5.947 9118956 PMC1169695

[B162] Rébeillé F. Neuburger M. Douce R. (1994). Interaction between glycine decarboxylase, serine hydroxymethyltransferase and tetrahydrofolate polyglutamates in pea leaf mitochondria. Biochem. J. 302, 223–228. 10.1042/bj3020223 7520695 PMC1137213

[B163] Rébeillé F. Ravanel S. Jabrin S. Douce R. Storozhenko S. Van Der Straeten D. (2006). Folates in plants: biosynthesis, distribution, and enhancement. Physiol. Plant. 126, 330–342. 10.1111/j.1399-3054.2006.00587.x

[B164] Reed L. S. Archer M. C. (1980). Oxidation of tetrahydrofolic acid by air. J. Agric. Food Chem. 28, 801–805. 10.1021/jf60230a044

[B165] Reyes-Hernandez B. J. Srivastava A. C. Ugartechea-Chirino Y. Shishkova S. Ramos-Parra P. A. Lira-Ruan V.. (2014). The root indeterminacy-to-determinacy developmental switch is operated through a folate-dependent pathway in *Arabidopsis thaliana*. New Phytol. 202, 1223–1236. 10.1111/nph.12757 24635769

[B166] Ringling C. Rychlik M. (2013). Analysis of seven folates in food by LC-MS/MS to improve accuracy of total folate data. Eur. Food Res. Technol. 236, 17–28. 10.1007/s00217-012-1849-x

[B167] Roje S. Chan S. Y. Kaplan F. Raymond R. K. Horne D. W. Appling D. R.. (2002a). Metabolic engineering in yeast demonstrates thats-adenosylmethionine controls flux through the methylenetetrahydrofolate reductase reaction *in vivo*. J. Biol. Chem. 277, 4056–4061. 10.1074/jbc.M110651200 11729203

[B168] Roje S. Janave M. T. Ziemak M. J. Hanson A. D. (2002b). Cloning and characterization of mitochondrial 5-formyltetrahydrofolate cycloligase from higher plants. J. Biol. Chem. 277, 42748–42754. 10.1074/jbc.M205632200 12207015

[B169] Rowe P. (1984). Folates in the Biosynthesis and Degradation of Purines. New York, NY: John Wiley.

[B170] Rychlik M. (2004). Revised folate content of foods determined by stable isotope dilution assays. J. Food Composit. Anal. 17, 475–483. 10.1016/j.jfca.2004.03.017

[B171] Rychlik M. Freisleben A. (2002). Quantification of pantothenic acid and folates by stable isotope dilution assays. J. Food Composit. Anal. 15, 399–409. 10.1006/jfca.2002.1081

[B172] Salbaum J. M. Kappen C. (2011). Diabetic embryopathy: a role for the epigenome? Birth Defects Res. A Clin. Mol. Teratol. 91, 770–780. 10.1002/bdra.20807 21538816 PMC3152694

[B173] Saxena C. Wang H. Kavakli I. H. Sancar A. Zhong D. (2005). Ultrafast dynamics of resonance energy transfer in cryptochrome. J. Am. Chem. Soc. 127, 7984–7985. 10.1021/ja0421607 15926801

[B174] Schnorr K. M. Nygaard P. Laloue M. (1994). Molecular characterization of *Arabidopsis thaliana* cDNAs encoding three purine biosynthetic enzymes. Plant J. 6, 113–121. 10.1046/j.1365-313X.1994.6010113.x 7920700

[B175] Scott J. M. (1984). Catabolism of folates, in Folates and Pterins, eds Blakley R. L. Benkovic S. J. (New York, NY: Wiley), 307–327.

[B176] Scott J. Rébeillé F. Fletcher J. (2000). Folic acid and folates: the feasibility for nutritional enhancement in plant foods. J. Sci. Food Agricul. 80, 795–824. 10.1002/(SICI)1097-0010(20000515)80:7<795::AID-JSFA599>3.0.CO;2-K

[B177] Shah S. Cossins E. (1970). Pteroylglutamates and methionine biosynthesis in isolated chloroplasts. FEBS Lett. 7, 267–270. 10.1016/0014-5793(70)80177-6 11947488

[B178] Shane B. (1989). Folylpolyglutamate synthesis and role in the regulation of one-carbon metabolism. Vitam. Hormon. 45, 263–335. 10.1016/S0083-6729(08)60397-0 2688305

[B179] Shimakawa T. Nieto F. J. Malinow M. R. Chambless L. E. Schreiner P. J. Szklo M. (1997). Vitamin intake: a possible determinant of plasma homocyst (e) ine among middle-aged adults. Ann. Epidemiol. 7, 285–293. 10.1016/S1047-2797(97)00004-5 9177112

[B180] Shingles R. Woodrow L. Grodzinski B. (1984). Effects of glycolate pathway intermediates on glycine decarboxylation and serine synthesis in pea (*Pisum sativum* L.). Plant Physiol. 74, 705–710. 10.1104/pp.74.3.705 16663485 PMC1066750

[B181] Sohn K. J. Stempak J. M. Reid S. Shirwadkar S. Mason J. B. Kim Y. I. (2003). The effect of dietary folate on genomic and p53-specific DNA methylation in rat colon. Carcinogenesis 24, 81–90. 10.1093/carcin/24.1.81 12538352

[B182] Sohta Y. Ohta T. Masada M. (1997). Purification and some properties of GTP cyclohydrolase I from spinach leaves. Biosci. Biotechnol. Biochem. 61, 1081–1085. 10.1271/bbb.61.1081

[B183] Song G. C. Choi H. K. Ryu C. M. (2013). The folate precursor para-aminobenzoic acid elicits induced resistance against Cucumber mosaic virus and *Xanthomonas axonopodis*. Ann. Bot. 111, 925–934. 10.1093/aob/mct049 23471007 PMC3631333

[B184] Song J. Sohn K. J. Medline A. Ash C. Gallinger S. Kim Y.-I. (2000). Chemopreventive effects of dietary folate on intestinal polyps in Apc+/− Msh2−/− mice. Cancer Res. 60, 3191–3199. 10866310

[B185] Spencer T. E. Jenster G. Burcin M. M. Allis C. D. Zhou J. Mizzen C. A.. (1997). Steroid receptor coactivator-1 is a histone acetyltransferase. Nature 389, 194–198. 10.1038/38304 9296499

[B186] Srivastava A. C. Ramos-Parra P. A. Bedair M. Robledo-Hernández A. L. Tang Y. Sumner L. W.. (2011). The folylpolyglutamate synthetase plastidial isoform is required for postembryonic root development in Arabidopsis. Plant Physiol. 155, 1237–1251. 10.1104/pp.110.168278 21233333 PMC3046582

[B187] Staben C. Rabinowitz J. (1984). Formation of formylmethionyl-tRNA and initiation of protein synthesis. Folates Pterins 1, 457–495.

[B188] Starkebaum G. Harlan J. M. (1986). Endothelial cell injury due to copper-catalyzed hydrogen peroxide generation from homocysteine. J. Clin. Invest. 77, 1370. 10.1172/JCI112442 3514679 PMC424498

[B189] Stea T. H. Johansson M. Jägerstad M. Frølich W. (2007). Retention of folates in cooked, stored and reheated peas, broccoli and potatoes for use in modern large-scale service systems. Food Chem. 101, 1095–1107. 10.1016/j.foodchem.2006.03.009

[B190] Stokes M. E. Chattopadhyay A. Wilkins O. Nambara E. Campbell M. M. (2013). Interplay between sucrose and folate modulates auxin signaling in Arabidopsis. Plant Physiol. 162, 1552–1565. 10.1104/pp.113.215095 23690535 PMC3707552

[B191] Storozhenko S. De Brouwer V. Volckaert M. Navarrete O. Blancquaert D. Zhang G. F.. (2007a). Folate fortification of rice by metabolic engineering. Nat. Biotechnol. 25, 1277–1279. 10.1038/nbt1351 17934451

[B192] Storozhenko S. Navarrete O. Ravanel S. De Brouwer V. Chaerle P. Zhang G. F.. (2007b). Cytosolic Hydroxymethyldihydropterin Pyrophosphokinase/Dihydropteroate Synthase from *Arabidopsis thaliana* a specific role in early development and stress response. J. Biol. Chem. 282, 10749–10761. 10.1074/jbc.M701158200 17289662

[B193] Strålsjö L. M. Witthöft C. M. Sjöholm I. M. Jägerstad M. I. (2003). Folate content in strawberries (Fragaria × ananassa): effects of cultivar, ripeness, year of harvest, storage, and commercial processing. J. Agric. Food Chem. 51, 128–133. 10.1021/jf020699n 12502396

[B194] Strandler H. S. Patring J. Jägerstad M. Jastrebova J. (2015). Challenges in the determination of unsubstituted food folates: impact of stabilities and conversions on analytical results. J. Agric. Food Chem. 63, 2367–2377. 10.1021/jf504987n 25642846

[B195] Strobbe S. Van Der Straeten D. (2017). Folate biofortification in food crops. Curr. Opin. Biotechnol. 44, 202–211. 10.1016/j.copbio.2016.12.003 28329726

[B196] Suh J. R. Herbig A. K. Stover P. J. (2001). New perspectives on folate catabolism. Annu. Rev. Nutr. 21, 255–282. 10.1146/annurev.nutr.21.1.255 11375437

[B197] Suzuki J. Y. Bollivar D. W. Bauer C. E. (1997). Genetic analysis of chlorophyll biosynthesis. Annu. Rev. Genet. 31, 61–89. 10.1146/annurev.genet.31.1.61 9442890

[B198] Suzuki Y. Brown G. M. (1974). The biosynthesis of folic acid XII. Purification and properties of dihydroneopterin triphosphate pyrophosphohydrolase. J. Biol. Chem. 249, 2405–2410. 4362677

[B199] Tomsho J. W. Moran R. G. Coward J. K. (2008). Concentration-dependent processivity of multiple glutamate ligations catalyzed by folylpoly-γ-glutamate Synthetase^†^. Biochemistry 47, 9040–9050. 10.1021/bi800406w 18672898 PMC2805413

[B200] Toth I. Lazar G. Goodman H. M. (1987). Purification and immunochemical characterization of a dihydrofolate reductase—thymidylate synthase enzyme complex from wild-carrot cells. EMBO J. 6, 1853–1858. 16453780 10.1002/j.1460-2075.1987.tb02443.x PMC553568

[B201] Triglia T. Cowman A. F. (1994). Primary structure and expression of the dihydropteroate synthetase gene of *Plasmodium falciparum*. Proc. Natl. Acad. Sci. U.S.A. 91, 7149–7153. 10.1073/pnas.91.15.7149 8041761 PMC44356

[B202] Triglia T. Cowman A. F. (1999). Plasmodium falciparum: a homologue of p-aminobenzoic acid synthetase. Exp. Parasitol. 92, 154–158. 10.1006/expr.1999.4400 10366540

[B203] Van Daele J. Blancquaert D. Kiekens F. Van Der Straeten D. Lambert W. E. Stove C. P. (2014). Folate Profiling in Potato (*Solanum tuberosum*) Tubers by Ultrahigh-Performance Liquid Chromatography-Tandem Mass Spectrometry. J. Agric. Food Chem. 62, 3092–3100. 10.1021/jf500753v 24655154

[B204] Van Wilder V. De Brouwer V. Loizeau K. Gambonnet B. Albrieux C. Van Der Straeten D.. (2009). C1 metabolism and chlorophyll synthesis: the Mg-protoporphyrin IX methyltransferase activity is dependent on the folate status. New Phytol. 182, 137–145. 10.1111/j.1469-8137.2008.02707.x 19076298

[B205] Von Wettstein D. Gough S. Kannangara C. G. (1995). Chlorophyll biosynthesis. Plant Cell. 7:1039. 10.1105/tpc.7.7.1039 12242396 PMC160907

[B206] Whiteley J. Drais J. Kirchner J. Huennekens F. M. (1968). Synthesis of 2-amino-4-hydroxy-6-formyl-7, 8-dihydropteridine and its identification as a degradation product of dihydrofolate. Arch. Biochem. Biophys. 126, 955–957. 10.1016/0003-9861(68)90490-6 5686605

[B207] Wilson S. D. Horne D. W. (1984). High-performance liquid chromatographic determination of the distribution of naturally occurring folic acid derivatives in rat liver. Anal. Biochem. 142, 529–535. 10.1016/0003-2697(84)90502-5 6442107

[B208] Wittek F. Kanawati B. Wenig M. Hoffmann T. Franz-Oberdorf K. Schwab W.. (2015). Folic acid induces salicylic acid-dependent immunity in Arabidopsis and enhances susceptibility to *Alternaria brassicicola*. Mol. Plant Pathol. 16, 616–622. 10.1111/mpp.12216 25348251 PMC6638506

[B209] Xu L. Glass C. K. Rosenfeld M. G. (1999). Coactivator and corepressor complexes in nuclear receptor function. Curr. Opin. Genet. Dev. 9, 140–147. 10.1016/S0959-437X(99)80021-5 10322133

[B210] Yoneyama T. Hatakeyama K. (1998). Decameric GTP cyclohydrolase I forms complexes with two pentameric GTP cyclohydrolase I feedback regulatory proteins in the presence of phenylalanine or of a combination of tetrahydrobiopterin and GTP. J. Biol. Chem. 273, 20102–20108. 10.1074/jbc.273.32.20102 9685352

[B211] Zhang G. F. Maudens K. E. Storozhenko S. Mortier K. A. Van Der Straeten D. Lambert W. E. (2003). Determination of total folate in plant material by chemical conversion into para-aminobenzoic acid followed by high performance liquid chromatography combined with on-line postcolumn derivatization and fluorescence detection. J. Agric. Food Chem. 51, 7872–7878. 10.1021/jf034808p 14690367

[B212] Zhang G. F. Storozhenko S. Van Der Straeten D. Lambert W. E. (2005). Investigation of the extraction behavior of the main monoglutamate folates from spinach by liquid chromatography-electrospray ionization tandem mass spectrometry. J. Chromatogr. A. 1078, 59–66. 10.1016/j.chroma.2005.04.085 16007982

[B213] Zhang H. Deng X. Miki D. Cutler S. La H. Hou Y. J.. (2012). Sulfamethazine suppresses epigenetic silencing in Arabidopsis by impairing folate synthesis. Plant Cell. 24, 1230–1241. 10.1105/tpc.112.096149 22447685 PMC3336112

[B214] Zhou H. R. Zhang F. F. Ma Z. Y. Huang H. W. Jiang L. Cai T.. (2013). Folate polyglutamylation is involved in chromatin silencing by maintaining global DNA methylation and histone H3K9 dimethylation in Arabidopsis. Plant Cell. 25, 2545–2559. 10.1105/tpc.113.114678 23881414 PMC3753382

[B215] Zrenner R. Stitt M. Sonnewald U. Boldt R. (2006). Pyrimidine and purine biosynthesis and degradation in plants. Annu. Rev. Plant Biol. 57, 805–836. 10.1146/annurev.arplant.57.032905.105421 16669783

